# Biotransformation of labdane and halimane diterpenoids by two filamentous fungi strains

**DOI:** 10.1098/rsos.170854

**Published:** 2017-11-08

**Authors:** Afif F. Monteiro, Cláudia Seidl, Vanessa G. P. Severino, Carmen Lúcia Cardoso, Ian Castro-Gamboa

**Affiliations:** 1 Núcleo de Bioensaios, Biossíntese e Ecofisiologia de Produtos Naturais (NuBBE), Universidade Estadual Paulista (UNESP), Instituto de Química, Departamento de Química Orgânica, Francisco Degni 55, Araraquara, 14800-900, Brazil; 2 Departamento de Química, Grupo de Cromatografia de Bioafinidade e Produtos Naturais, Faculdade de Filosofia, Ciências e Letras de Ribeirão Preto, Universidade de São Paulo, Ribeirão Preto, 14040-901, São Paulo, Brazil; 3 Universidade Federal de Goiás (UFG), Instituto de Química, Campus Samambaia, Goiânia, 74690-900, Brazil

**Keywords:** labdane and halimane diterpenoids, whole-cell biotransformation, anticholinesterase inhibitors, *Fusarium oxysporum*, *Myrothecium verrucaria*

## Abstract

Biotransformation of natural products by filamentous fungi is a powerful and effective approach to achieve derivatives with valuable new chemical and biological properties. Although diterpenoid substrates usually exhibit good susceptibility towards fungi enzymes, there have been no studies concerning the microbiological transformation of halimane-type diterpenoids up to now. In this work, we investigated the capability of *Fusarium oxysporum* (a fungus isolated from the rhizosphere of *Senna spectabilis*) and *Myrothecium verrucaria* (an endophyte) to transform halimane (**1**) and labdane (**2**) acids isolated from *Hymenaea stigonocarpa* (Fabaceae). Feeding experiments resulted in the production of six derivatives, including hydroxy, oxo, formyl and carboxy analogues. Incubation of **1** with *F. oxysporum* afforded 2-oxo-derivative (**3**), while bioconversion with *M. verrucaria* provided 18,19-dihydroxy (**4**), 18-formyl (**5**) and 18-carboxy (**6**) bioproducts. Transformation of substrate **2** mediated by *F. oxysporum* produced a 7*α*-hydroxy (**7**) derivative, while *M. verrucaria* yielded 7*α*- (**7**) and 3*β*-hydroxy (**8**) metabolites. Unlike *F. oxysporum*, which showed a preference to transform ring B, *M. verrucaria* exhibited the ability to hydroxylate both rings A and B from substrate **2**. Additionally, compounds **1**–**8** were evaluated for inhibitory activity against Hr-AChE and Hu-BChE enzymes through ICER-IT-MS/MS assay.

## 1. Introduction

Diterpenoids have attracted outstanding attention because many of these constituents display a wide variety of pronounced biological activities, following the diversity of new structures discovered each year. These metabolites are known to be produced mostly from plants, but they have also been found from microorganisms, such as fungi and bacteria, as well as marine organisms [1–3]. Labdanes constitute a large group of diterpenoids, having a chemical framework of C-20 skeleton comprising a decalin core and a C-6 side chain, cyclic or aliphatic. They usually exhibit five stereocentres and can occur in both normal and antipodal series [4]. Halimanes arise from labdanes by migration of C-20 methyl group from C-10 to C-9 position. Among plants producing diterpenoids as regular constituents, Fabaceae deserves to be highlighted. Remarkably, it comprises *Hymenaea* genus, a small group of about 14 species, which are rich sources of *ent*-labdane and *ent*-halimane diterpenoids [5].

In a previous work [6], we reported the isolation of (+)-(4*R*, 5*S*, 8*R*, 9*S*)-18-hydroxy-*ent*-halima-1(10),13-(*E*)-dien-15-oic (**1**) and (+)-(5*S*, 8*S*, 9*R*, 10*S*)-lab-13-en-8*β*-ol-15-oic (**2**) acids from the ethanol extract of flowers and leaves of *H. stigonocarpa*, respectively, as major constituents. Herein, substances **1** and **2** were reisolated and assayed for the inhibitory activity of human recombinant acetyl (Hr-AChE) and human serum butyrylcholinesterase (Hu-BChE) enzymes, key therapeutic and diagnostic targets for Alzheimer's disease [7]. However, these metabolites displayed weak inhibitory activity on target enzymes, which motivated us to carry out biotransformation experiments in order to generate new structurally related and potentially bioactive analogues.

Biotransformation mediated by filamentous fungi consists of a powerful method to perform chemical modifications of a variety of starting materials such as bioactive natural products, to obtain derivatives with improved biological properties or even new biological activities [8]. This approach stands out as a promising alternative to conventional chemical methods since fungi contain multi-enzymatic systems with broad specificities and are, therefore, able to catalyse chemo, regio and stereoselective reactions on non-activated molecular sites that are normally unreactive or difficult to reach chemically [9]. Furthermore, microbial transformation is a fast, efficient, cost-effective and ecologically friendly technique because it requires only mild reaction conditions [10].

Although several biotransformation studies of labdane-skeleton have been reported so far [1,8], to the best of our knowledge, there have been no reports on literature concerning biotransformation of halimane-type, even though halimanes are closely related to labdanes. In this work, we report the investigation of the biotransformation-promoting capabilities of compounds **1** and **2** by two filamentous fungi, namely *Fusarium oxysporum* and *Myrothecium verrucaria*, together with the inhibitory activity of enzymes Hr-AChE and Hu-BChE of starting compounds and their biotransformation products.

## 2. Results and discussion

### 2.1 Determination of biotransformed products

**Figure 1.**
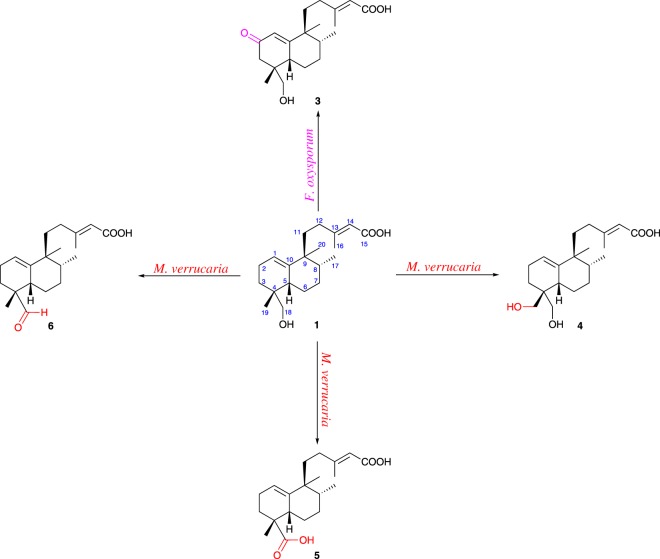
Biotransformation products of halimane diterpenoid **1** obtained with *F. oxysporum* and *M. verrucaria*.

Biotransformation of **1** by *F. oxysporum* afforded one structurally related compound **3** (figure 1), while *M. verrucaria* provided three closely related derivatives, metabolites **4**, **5** and **6** (figure 1). Compound **3** was obtained as a white and amorphous solid. Its molecular formula was determined as C_20_H_30_O_4_ based on its HR-ESI-MS spectrum, which revealed an ion at *m/z* 333.2082 [M − H]^−^ (calcd. for C_20_H_29_O_4_, 333.2071), suggesting substrate suffered loss of two hydrogen atoms and insertion of an oxygen, when compared to the molecular formula of starting compound (C_20_H_32_O_3_). ^1^H and ^13^C NMR spectra were quite similar to those of **1**, allowing direct assignments based on comparison (table 1). The main differences between **1** and **3** corresponded to the presence of signals arising from the resonance of a methylene group adjacent to an *α*,*β*-unsaturated carbonyl group (*δ*_H_ 2.37 and 1.92), and deshielding of H-1 signal from 5.37 ppm for **1** to *δ*_H_ 5.83 for **3**. ^13^C NMR spectrum confirmed the presence of a signal corresponding to a typical resonance of a keto group (*δ*_C_ 202.1), which was assigned to C-2. This assignment was further supported by *β*-effects observed at C-1 (+3.7) and C-3 (+15.6), and confirmed by HMBC correlation (see electronic supplementary material) from H_2_-3 (*δ*_H_ 2.37; 1.92) to C-2. Therefore, biotransformation product **3** is the 2-oxo-derivative of substrate **1**.

**Table 1.** NMR spectroscopic data of compounds **3** in CD_3_OD^a^, **4** and **6** in CDCl_3_^a^.

	Comp. **3**		Comp. **4**		Comp. **6**	
position	*δ*_C_	*δ*_H_, mult. (*J* in Hz)	*δ*_C_	*δ*_H_, mult. (*J* in Hz)	*δ*_C_	*δ*_H_, mult. (*J* in Hz)
1	125.1	5.83, *s*	120.8	5.35, *s*	121.0	5.43, *t* (3.3; 6.5)
2	202.1		22.3	2.04, *m*	22.1	2.19, *m*
						2.07, *m*
3	43.2	2.37, *d* (16.0)	22.8	1.37, *m*	25.2	1.45, *m*
		1.92, *d* (16.0)				1.36, *m*
4	39.8		39.7		47.6	
5	43.7	2.46, *dd* (4.4, 12.6)	35.4	2.06, *m*	40.4	2.08, *m*
6	25.5	1.95, *m*	23.9	1.60, *m*	25.2	1.73, *m*
		1.62, *qd* (4.4, 12.6, 20.6)		1.32, *m*		1.45, *m*
7	29.5	2.17, *m*	29.4	2.03, *m*	28.9	2.00, *m*
		1.47, *m*		1.39, *m*		1.36, *m*
8	42.8	1.89, *m*	39.8	1.58, *m*	39.3	1.60, *m*
9	46.7		43.6		43.3	
10	173.7		140.8		139.5	
11	38.4	2.31, *td* (3.5, 13.1; 22.7)	37.1	2.13, *m*	36.9	2.09, *m*
		1.52, *m*		1.26, *m*		1.33, *m*
12	36.7	2.14, *m*	36.4	2.02, *m*	36.3	2.06, *m*
		1.77, *td* (3.5, 13.1; 22.7)		1.84, *m*		1.84, *m*
13	159.2		164.4		164.8	
14	117.9	5.69, *s*	114.7	5.69, *s*	114.7	5.68, *s*
15	171.9		171.0		171.2	
16	19.0	2.12, *s*	19.4	2.14, *s*	19.6	2.18, *d* (1.1)
17	15.9	0.87, *d* (7.1)	15.7	0.82, *d* (7.0)	15.7	0.83, *d* (7.0)
18	68.8	3.56, *d* (11.0)	68.1	3.60, *d* (9.6)	207.5	9.65, *s*
		3.30, *d* (11.0)		3.70, *d* (9.6)		
19	23.2	1.06, *s*	69.5	3.65, *bs*	19.9	1.07, *s*
20	21.4	1.09, *s*	22.0	0.91, *s*	22.3	0.95, *s*

^a^Run at 600.13 MHz.

Metabolite **4** showed molecular formula as C_20_H_32_O_4_, *m/z* 335.2231 [M − H]^−^ (calcd. for C_20_H_31_O_4_, 335.2227), indicating incorporation of one additional oxygen atom into the substrate, and loss of one hydrogen atom. Its ^1^H NMR spectrum (table 1) showed a signal at *δ*_H_ 3.65 (*bs*) arising from a new oxymethylene group. The ^13^C chemical shift was assigned to be 69.5 through direct correlation by HSQC experiment and the position of hydroxylation occurrence was established at C-19 by HMBC correlations from H_2_-18 (*δ*_H_ 3.70; 3.60) to the signals at *δ*_C_ 35.4 (C-5) and 68.1 (C-18). In addition, a *β*-effect was observed at C-4 (+3.7), while *γ*-effects were noticed at C-3 (−3.7) and C-5 (−5.1), corroborating the proposal of an oxidation in this position. Spectroscopic data of compound **5** (table 1) suggested an 18-carboxy analogue, which is in agreement with those data reported for its dimethyl ester [11], previously isolated from the aerial parts of *Halimium viscosum*.

Natural analogue **6** gave the molecular formula C_20_H_30_O_3_, *m/z* 317.2125 [M − H]^−^ (calcd. for C_20_H_29_O_3_, 317.2122), suggesting that an oxidation took place by the loss of two hydrogen atoms. ^1^H NMR spectrum of **6** (table 1) showed a signal at 9.65 (*s*) assigned to an aldehyde hydrogen, pinpointing an oxidation at C-18. Location of formyl group was confirmed by HSQC correlation between *δ*_H_ 9.65 and the signal at *δ*_C_ 207.5 and through HMBC correlations observed among H_3_-19 (*δ*_H_ 1.07) and C-4 (*δ*_C_ 47.6), and *δ*_C_ 207.5 (C-18). Additionally, *β*-effect of C-4 (+11.6) and *γ*-effects of C-3 and C-5 were verified, which are quite consistent with this proposal.

**Figure 2.**
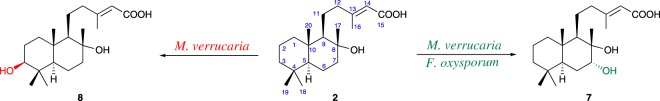
Derivatives produced by biotransformation of diterpenoid **2** with *F. oxysporum* and *M. verrucaria*.

**Figure 3.**
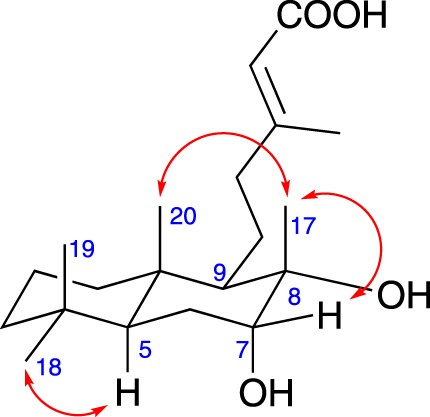
Key NOESY correlations observed for compound **7**.

Biotransformation of **2** with *F. oxysporum* provided compound **7**, while *M. verrucaria* afforded metabolites **7** and **8** (figure 2). Compound **7** showed molecular formula C_20_H_34_O_4_, *m/z* 337.2391 [M − H]^−^ (calcd. for C_20_H_33_O_4_, 337.2384), indicating the addition of an oxygen atom into the substrate by the microorganisms, when compared to the molecular formula of **2** (C_20_H_34_O_3_). Inspection of uni- and bi-dimensional NMR spectroscopic data (table 2) revealed a dihydroxy analogue. Location of the second hydroxyl group was established as adjacent to C-8, at position C-7, based on correlation of H-7 (*δ*_H_ 3.58, *t*, *J* = 2.6, 5.2 Hz) with the resonance corresponding to C-8 (*δ*_C_ 75.7). ^13^C NMR data analysis of metabolite **7** compared to that of **2** showed consistent effects, namely *β*-effects of C-8 (+0.7) and C-6 (+6.1), and *γ*-effects of C-9 (−6.4) and C-5 (−9.7). *α*-Orientation of the hydroxyl group at C-7 was determined by *g*NOESY interactions (figure 3), multiplicity and magnitude of the coupling constants. Therefore, biotransformation product **7** exhibits a 7*α*,8*α*-diol system. Spectroscopic data of compound **8** were quite similar to those of its methyl ester derivative obtained from the resin of *Acacia* sp. [12].

**Table 2.** NMR Data of Compound **7** (CD_3_OD, 600.13 MHz).

position	*δ*_C_, type	*δ*_H_ (*J* in Hz)	HMBC
1	40.9, CH_2_	1.66, *m*	
		1.02, *m*	
2	19.5, CH_2_	1.66, *m*	
		1.46, *m*	
3	43.2, CH_2_	1.40, *m*	
		1.21, *m*	
4	36.7, C		
5	47.7, CH	1.52, *m*	20, 18, 6, 19, 7
6	27.6, CH_2_	1.79, *m*	
		1.60, *m*	
7	75.5, CH	3.58, *t_ap_* (2.6, 5.2)	17, 9, 5, 8
8	75.7, C		
9	56.1, CH	1.38, *m*	20, 17, 10, 12, 8
10	40.0, C		
11	24.6, CH_2_	1.60, *m*	
		1.40, *m*	
12	45.4, CH_2_	2.33, *m*	16, 9, 14, 13
		2.19, *m*	
13	162.1, C		
14	116.7, CH	5.67, *bs*	16, 12
15	170.9, C		
16	19.1, CH_3_	2.14, *s*	12, 14, 13, 15
17	23.1, CH_3_	1.12, *s*	9, 8
18	22.0, CH_3_	0.82, *s*	19, 3, 5
19	33.6, CH_3_	0.87, *s*	18, 5
20	15.6, CH_3_	0.84, *s*	1, 5, 9

*Fusarium oxysporum* is a filamentous fungus that has been successfully applied to biotransformation of substrates belonging to the classes of monoterpenoids [13], diterpenoids [14] and steroids [15]. Therefore, conversion of substrates **1** and **2** into products **3** and **7**, respectively, suggests oxidative reactions mediated by cytochrome P450 monooxygenase [9] at C-2 (substrate **1**) and C-7 (substrate **2**), and subsequent oxidation of 2-hydroxy derivative into its 2-oxo product.

Oxidation of substrate **1** at C-2 to provide **3** is probably favoured because these hydrogens are allylic to 1,10-double bond. Previously microbial transformations of diterpenoid substrates performed by *Fusarium* species encompasses conversion of dehydroabietic acid into its 1*β*-hydroxy derivative by *F. oxysporum* [14], modification of sclareol with *F. lini* leading to 1*β*-hydroxy and (12*S*)-12-hydroxysclareol derivatives [16], and oxidation of cupressic acid by *F. graminearum* to produce four metabolites, including 3*β*-hydroxy and 7*α*-hydroxy analogues [17]. Sequential oxidations at position C-2 from diterpenoid substrate to alcohol and then oxo derivatives have been found to occur only with another fungus, *Mucor plumbeus* [18].

Only one report involving biotransformation process with *Myrothecium* species, namely *M. roridum*, was found in the literature, however, dedicated to malachite green (a triphenylmethane dye) decolorization [19]. Despite the lack of reference, it is well established that fungi can catalyse a series of transformations through their enzymatic machinery and diterpenoids exhibit good susceptibility towards fungal enzymes. Thus, the formation of hydroxy derivatives **4** (C-19), **7** (C-7) and **8** (C-3) by *M. verrucaria* would also involve the action of P450 monooxygenase, while analogues **5** and **6** would comprise carboxylation of substrates at C-18, to formyl and carboxy products, subsequently.

Oxidations in substrate **1** were observed only in ring A by both microorganisms. However, *F. oxysporum* had been able to oxidize just position C-7 (**7**) from substrate **2** showing preference by ring B, while *M. verrucaria* not only provided the same derivative but also afforded a second 3-hydroxy derivative (**8**, oxidized in ring A), indicating no specific ring preference. Only the yields of compound **7** could be compared as it was commonly produced by both fungi, showing a higher yield from *F. oxysporum*, despite *M. verrucaria* deviates substrate for conversion between two derivatives.

### 2.2 Anticholinesterase assays

Labdane-type diterpenoids [20] and semisynthetic labdane derivatives [21] are reported to exhibit significant anticholinesterase activities. In this context, compounds **1**–**8** were screened for inhibitor candidates by means of hydrolysis of acetylcholine (ACh) through on-flow screening by Hr-AChE and Hu-BChE-ICER-IT-MS/MS assay (table 3). In this study, substrates **1** and **2**, and their biotransformation derivatives demonstrated different inhibitory activities relative to the structural modifications.

**Table 3.** AChE and BChE inhibitory activity (% inhibition ± s.e.m) and IC_50_ values of compounds **1**–**8**.

	(% inhibition ± s.e.m.^a^) at 100 µM		
comp.	ICER-AChE_hr_	ICER-BChE_hu_	IC_50_ (µM) AChE_hr_
1	24.96 ± 0.04	36.09 ± 0.28	>100
2	26.47 ± 3.10	13.26 ± 0.39	>100
3	30.13 ± 0.30	14.47 ± 2.47	>100
4	39.40 ± 3.86	14.52 ± 2.42	>100
5	48.63 ± 0.84	8.17 ± 1.32	>100
6	3.23 ± 1.7	25.07 ± 0.14	>100
7	4.71 ± 0.11	20.07 ± 1.73	>100
8	54.14 ± 0.90	13.33 ± 2.62	95.74 ± 1.7
Gal^b^	93.31 ± 1.28	75.85 ± 1.74	—

^a^Standard error mean (*n* = 3).

^b^Galanthamine—Reference for AChE and BChE inhibition.

The oxidations occurred into substrate **1** gradually increased the inhibition potential of compounds **3**, **4** and **5** over enzyme Hr-AChE, at the same time they selectively decreased their activity towards Hu-BChE. Compound **5**, bearing an 18-carboxy-substituent was found to be the most active halimane derivative against Hr-AChE, displaying inhibition around 50%. However, substance **6**, possessing an 18-formyl group, showed the worse inhibition over the same enzyme.

Hydroxylation of **2** (ring B) was observed to drastically decrease the Hr-AChE inhibition of compound **7**, which possesses a 7,8-diol system, while insertion of the 3-hydroxy group (ring A) in derivative **8** showed to selectively improve its Hr-AChE inhibition. Interestingly, these modifications did not affect Hu-BChE activity to an appreciable extent.

The correlations inferred between structure modifications and resulting inhibitory activities of compounds **1** to **8** suggests oxo, hydroxyl, formyl and carboxy substituents play determinant roles in increasing selectivity and potency of these compounds over Hr-AChE since they share similar hydrocarbon backbones, while these groups are responsible for opposite effects towards Hu-BChE. Such findings indicate oxygen atoms, from substituents added to the bioproducts, could be able to interact with Hr-AChE mainly through hydrogen bonds, possibly formed between hydroxyl and carbonyl groups and the residues from the catalytic triad of Hr-AChE active site [21].

As screening results, two hits were found for Hr-AChE, derivatives **5** and **8**, with inhibition percentage around 50% in comparison to standard inhibitor galanthamine. However, despite oxidations at position C-18 (**5**) and at C-3 (**8**) having increased the inhibitory potential of these derivatives in comparison to the substrates **1** and **2**, respectively, compound **5** can be considered to be inactive based on its IC_50_ value (greater than 100 µM), whereas **8** was only weakly active (IC_50_ = 95.74 µM), showing some enhanced activity but still requiring further structure optimization for better potency.

## 3. Experimental

### 3.1. General experimental procedures

Optical rotations were measured on a Schimdt-Haensh (Berlin, Germany) Polartronic H-100 polarimeter using quartz cells of 1 dm path length, at 25°C. IR spectra were recorded on a FT-IR Vertex 70 Bruker spectrometer, operating in ATR mode. NMR spectra were acquired on a Bruker Avance III HD 600 spectrometer (14.1 T—600.13 MHz for ^1^H and 150.9 for ^13^C) equipped with a Triple Inverse TCI Cryo-Probehead (5.0 mm). Chloroform-*d*_1_ and methanol-*d*_4_ were used as solvents. Chemical shifts (*δ*) are given in ppm and coupling constants (*J*) in hertz (Hz). High-resolution electrospray ionization mass spectra (HR-ESI-MS) were obtained on a Bruker Daltonics Inc Q-TOF Maxis Impact mass spectrometer (Billerica, MA, USA) in negative ion mode, employing sodium formate (HCOONa) as internal standard. Chromatographic separations were performed on a Shimadzu Prominence HLPC system (Kyoto, KY, Japan) composed of following modules: two LC-6AD pumps, DGU-20A degasser unit, SIL-10AF auto sampler, SPD-20A Diode Array Detector (set at 210 and 254 nm) and CBM-20A Communication Bus Module; controlled by Lab Solutions software. High-performance liquid chromatography (HPLC) columns used were analytical Kinetex® (5 µm, C_18_, 100 Å, h: 150 × 4.60 mm) and semi-preparative Kinetex® (5 µm, C_18_, 100 Å, h: 250 × 10.0 mm)—Phenomenex (Torrance, CA, USA). Solvents used were HPLC-grade from J.T.Baker®—Avantor (Center Valley, PA, USA).

### 3.2. Plant material

Flowers and leaves of *H. stigonocarpa* were collected during flowering stage in Catalão, Brazil. A voucher specimen has been deposited at EMBRAPA—Recursos Genéticos e Biotecnologia Herbarium, under no. GD046.

### 3.3. Extraction and isolation of substrates 1 and 2

Fractionation of 10.0 g of each ethanol extract from the flowers and leaves of *H. stigonocarpa* according to the procedure described by Monteiro *et al.* [6] afforded 0.6 g of compound **1** and 0.35 g of compound **2**, respectively. Physical and spectroscopic data were compared with those reported for both compounds and showed full agreement.

(+)-(4*R*, 5*S*, 8*R*, 9*S*)-18-Hydroxy-*ent*-halima-1(10),13-(*E*)-dien-15-oic acid (**1**): white, amorphous solid, [α]_D_^25 ^+ 90.0 (*c* 0.1, MeOH); ^1^H NMR (CD_3_OD, 600.13 MHz): *δ*_H_ 5.66 (1H, *s*, H-14), 5.37 (1H, *t*, *J* = 3.5, 6.9 Hz, H-1), 3.43 (1H, *d*, *J* = 10.7 Hz, H-18a), 3.23 (1H, *t*, *J* = 10.7 Hz, H-18b), 2.19 (1H, *td*, *J* = 3.9, 13.0 Hz, H-11a), 2.10 (3H, *s*, H-16), 2.08 (2H, *m*, H-2), 2.07 (1H, *m*, H-7a), 2.03 (1H, *m*, H-12a), 1.90 (1H, *dd*, *J* = 3.3, 9.2 Hz, H-5), 1.82 (1H, *td*, *J* = 3.9, 13.0 Hz, H-12b), 1.66 (1H, *m*, H-6a), 1.58 (1H, *m*, H-8), 1.35 (1H, *m*, H-3a), 1.33 (1H, *m*, H-7b), 1.28 (1H, *m*, H-6b), 1.24 (1H, *m*, H-11b), 1.08 (1H, *dt*, *J* = 4.2, 8.3, 12.4 Hz, H-3b), 0.96 (3H, *s*, H-19), 0.95 (3H, *s*, H-20), 0.85 (3H, *d*, *J* = 7.0 Hz, H-17); ^13^C NMR (CD_3_OD, 150.9 MHz): *δ*_C_ 170.4 (C, C-15), 162.8 (C, C-13), 142.5 (C, C-10), 121.4 (CH, C-1), 116.3 (CH, C-14), 70.1 (CH_2_, C-18), 44.4 (C, C-9), 41.8 (CH, C-5), 41.1 (CH, C-8), 38.5 (CH_2_, C-11), 37.2 (CH_2_, C-12), 36.8 (C, C-4), 30.4 (CH_2_, C-7), 27.6 (CH_2_, C-3), 25.2 (CH_2_, C-6), 23.4 (CH_2_, C-2), 22.5 (CH_3_, C-20), 22.3 (CH_3_, C-19), 19.1 (CH_3_, C-16), 16.0 (CH_3_, C-17).

(+)-(5*S*, 8R, 9*R*, 10*S*)-Lab-13-en-8*α*-ol-15-oic acid (**2**): white, amorphous solid, [α]_D_^25 ^+ 23.0 (*c* 0.1, MeOH); ^1^H NMR (CD_3_OD, 600.13 MHz): *δ*_H_ 5.67 (1H, *s*, H-14), 2.33 (1H, *td*, *J* = 5.1, 12.8 Hz, H-12a), 2.17 (1H, *td*, *J* = 5.1, 12.8 Hz, H-12b), 2.14 (3H, *d*, *J* = 1.3 Hz, H-16), 1.83 (1H, *dt*, *J* = 3.1, 6.2, 12.4 Hz, H-7a), 1.67 (1H, *m*, H-1a), 1.66 (1H, *m*, H-2a), 1.66 (1H, *m*, H-6a), 1.63 (1H, *m*, H-11a), 1.46 (1H, *m*, H-7b), 1.45 (1H, *m*, H-2b), 1.38 (1H, *m*, H-3a), 1.37 (1H, *m*, H-11b), 1.32 (1H, *m*, H-6b), 1.19 (1H, *td*, *J* = 4.1, 13.5 Hz, H-3b), 1.14 (3H, *d*, *J* = 0.6 Hz, H-17), 1.12 (1H, *t*, *J* = 3.9, 7.8 Hz, H-9), 0.98 (1H, *m*, H-1b), 0.96 (1H, *m*, H-5), 0.89 (3H, *s*, H-19), 0.85 (3H, *s*, H-20), 0.82 (3H, *s*, H-18); ^13^C NMR (CD_3_OD, 150.9 MHz): *δ*_C_ 170.7 (C, C-15), 162.6 (C, C-13), 116.3 (CH, C-14), 75.0 (C, C-8), 62.5 (CH, C-9), 57.4 (CH, C-5), 45.5 (CH_2_, C-12), 45.0 (CH_2_, C-7), 43.1 (CH_2_, C-3), 41.1 (CH_2_, C-1), 40.4 (C, C-10), 34.2 (C, C-4), 33.9 (CH_3_, C-19), 25.0 (CH_2_, C-11), 23.8 (CH_3_, C-17), 21.9 (CH_3_, C-18), 21.5 (CH_2_, C-6), 19.5 (CH_2_, C-2), 19.2 (CH_3_, C-16), 16.10 (CH_3_, C-20).

### 3.4. Microorganisms and culture conditions

Cultures of *Myrothecium verrucaria* (AJ302003.1) were isolated from healthy leaves of a *Senna spectabilis* (Fabaceae) specimen, collected around Institute of Chemistry—UNESP, Araraquara, Brazil, by Dr Lisinéia M. Zanardi under the supervision of Prof. Ângela R. Araújo. A voucher specimen of *S. spectabilis* has been deposited at Herbário do Jardim Botânico de São Paulo, under no. SP384109. *Fusarium oxysporum* (HM346538.1) cultures were isolated from the rhizosphere of *S. spectabilis* seedlings cultivated in the hydroponic medium by Dr Patrícia Cardoso, supervised by Prof. Ian Castro-Gamboa. Voucher samples of *M. verrucaria* (Cs-f23) and *F. oxysporum* (CSP-30) are maintained in the Collection ‘Micoteca’ at NuBBE, Department of Organic Chemistry, Institute of Chemistry, São Paulo State University, Araraquara, Brazil. Both fungi were pre-cultivated in Petri dishes containing Potato Dextrose Agar (PDA) medium for 7 days, at 28°C, prior to liquid medium inoculation.

### 3.5. Biotransformation procedure

Both strains were cultured in a two-step procedure. Firstly, a spore suspension of each fungus was inoculated into three 500 ml Erlenmeyer flasks containing 250 ml of sterile CZAPEK Broth (pH ∼ 5.3), and subsequently, incubated at 110 r.p.m. for 72 h, at 28°C. Then, the mycelial mass was filtered, inoculated in fresh CZAPEK media (triplicate for substrate **1** and duplicate for **2**) and reincubated in same shake conditions for additional 144 h. Substrates were dissolved in dimethyl sulfoxide (DMSO) at a concentration of 50.0 mg ml^−1^ and added to the flask to achieve a final concentration of 0.2 mg ml^−1^. Culture controls consisted of three flasks containing CZAPEK medium in which fungi were individually grown in the presence of the same amount of DMSO but absence of substrate. Substrate controls comprised medium and the same concentration of starting compounds, without strains. Controls were incubated in identical conditions and simultaneously to biotransformation experiments. In order to monitor the consumption of substrates as well as detect the formation of biotransformation products, samples of 1 ml of medium were collected daily during incubation course, extracted with ethyl acetate (AcOEt), and analysed by HPLC-DAD (conditions specified in the next section).

### 3.6. Extraction, HPLC analyses and separations of extracts

Mycelia of *F. oxysporum* and *M. verrucaria* from experiments with both substrates were filtered and extracted using AcOEt. The solvent was removed under reduced pressure to afford four extracts: *F. oxysporum*—diterpenoid substrate **1** (FoD1), 108.0 mg; *M. verrucaria*—substrate **1** (MvD1), 117.0 mg; *F. oxysporum*—substrate **2** (FoD2), 74.9 mg; and *M. verrucaria*—substrate **2** (MvD2), 72.8 mg. Analytical-scale HPLC analyses of extracts were carried out in linear gradient elution mode, with a mobile phase composed by acetonitrile-water (0.1% formic acid), ranging from 25 : 75 (v/v) to 0 : 100 (v : v) within 20 min and held for additional 5 min. Flow rate was 1.00 ml min^−1^ and the injection volume was 20 µl from a solution of concentration 5.0 mg ml^−1^. Analytical-scale conditions were transposed to semi-preparative scale by employing a 3.94 ml min^−1^ flow rate, 40 min running time and injection volume of 50 µl (25 mg ml^−1^).

### 3.7. Isolation of biotransformation products from diterpenoid 1

Fractionation of extract FoD1 by semi-preparative HPLC rendered 41 fractions and fraction FoD1.8 afforded compound **3** (2.0 mg, 8%). Chromatographic separation of extract MvD1 resulted in 26 fractions, of which fraction MvD1.15 yielded compound **4** (1.0 mg, 4%), fraction MvD1.19 afforded compound **5** (2.4 mg, 9.6%) and fraction MvD1.23 provided compound **6** (0.8 mg, 3.2%).

(+)-(4*R*, 5*S*, 8*R*, 9*S*)-2-Oxo-18-hydroxy-*ent*-halima-1(10),13-(*E*)-dien-15-oic acid (**3**): white, amorphous solid, [α]_D_^25 ^+ 63.5 (*c* 0.1, MeOH); IR ν_max_ 3390, 2902, 1700, 1650, 1040 cm^−1^; ^1^H and ^13^C NMR data, see table 1; HR-ESI-MS *m/z* 333.2082 [M − H]^−^ (calcd. for C_20_H_29_O_4_ 333.2071).

(+)-(5*S*, 8*R*, 9*S*)-18,19-Dihydroxy-*ent*-halima-1(10),13-(*E*)-dien-15-oic acid (**4**): white, amorphous solid, [α]_D_^25 ^+ 28.3 (*c* 0.1, MeOH); IR ν_max_ 3380, 2930, 1680, 1640, 1010 cm^−1^; ^1^H and ^13^C NMR data, see table 1; HR-ESI-MS *m/z* 335.2231 [M − H]^−^ (calcd. for C_20_H_31_O_4_ 335.2227).

(+)-(4*R*, 5*S*, 8*R*, 9*S*)-18-Carboxy-*ent*-halima-1(10),13-(*E*)-dien-15-oic acid (**5**): white, amorphous solid, [α]_D_^25^ + 12.1 (*c* 0.1, MeOH); IR ν_max_ 2910, 1700, 1660, 1045 cm^−1^; ^1^H NMR (CDCl_3_, 600.13 MHz): *δ*_H_ 5.69 (1H, *bs*, H-14), 5.33 (1H, *d*, *J* = 4.9 Hz, H-1), 2.19 (3H, *d*, *J* = 1.0 Hz, H-16), 2.18 (1H, *m*, H-11a), 2.16 (1H, *m*, H-2a), 2.10 (1H, *m*, H-5), 2.09 (1H, *m*, H-12a), 2.08 (1H, *m*, H-7a), 2.06 (1H, *m*, H-2b), 1.84 (1H, *m*, H-12b), 1.83 (1H, *m*, H-3a), 1.61 (1H, *m*, H-8), 1.54 (1H, *m*, H-3b), 1.45 (2H, *m*, H-6), 1.38 (1H, *m*, H-7b), 1.28 (1H, *m*, H-11b), 1.25 (3H, *s*, H-19), 0.95 (3H, *s*, H-20), 0.83 (3H, *d*, *J* = 6.9 Hz, H-17); ^13^C NMR (CDCl_3_, 150.9 MHz): *δ*_C_ 184.0 (C, C-18), 172.2 (C, C-15), 164.7 (C, C-13), 139.5 (C, C-10), 119.9 (CH, C-1), 114.9 (CH, C-14), 44.5 (C, C-4), 43.6 (C, C-9), 41.6 (CH, C-5), 39.9 (CH, C-8), 37.0 (CH_2_, C-11), 36.3 (CH_2_, C-12), 29.4 (CH_2_, C-7), 26.6 (CH_2_, C-6), 24.3 (CH_2_, C-3), 22.3 (CH_2_, C-2), 22.1 (CH_3_, C-19), 21.9 (CH_3_, C-20), 19.7 (CH_3_, C-16), 15.6 (CH_3_, C-17); HR-ESI-MS *m/z* 333.2081 [M − H]^−^ (calcd. for C_20_H_29_O_4_ 333.2071).

(+)-(4*R*, 5*S*, 8*R*, 9*S*)-18-Formyl-*ent*-halima-1(10),13-(*E*)-dien-15-oic acid (**6**): white, amorphous solid, [α]_D_^25^ + 4.7 (*c* 0.1, MeOH); IR ν_max_ 2930, 1700, 1640 cm^−1^; ^1^H and ^13^C NMR data, see table 1; HR-ESI-MS *m/z* 317.2125 [M − H]^−^ (calcd. for C_20_H_29_O_3_ 317.2122).

### 3.8. Isolation of biotransformation products from diterpenoid 2

Extract FoD2 was chromatographed to give 24 fractions and fraction FoD2.12 afforded compound **7** (0.6 mg, 2.4%). Finally, fractionation of extract MvD2 rendered 23 fractions, and fraction MvD2.16 showed to be identical to that of compound **7** (0.4 mg, 1.6%), while fraction MvD2.7 provided compound **8** (1.5 mg, 6.4%).

(-)-(5S*, 7R*, 8R*, 9R*, 10S*)-Lab-13-en-7*α*,8*α*-diol-15-oic acid (**7**): pale yellow, amorphous solid; [α]_D_^25^ -2.2 (*c* 0.1, MeOH); IR ν_max_ 3380, 2905, 1685, 1050 cm^−1^; ^1^H and ^13^C NMR data, see table 3; HR-ESI-MS *m/z* 337.2391 [M − H]^−^ (calcd. for C_20_H_33_O_4_ 337.2384).

(+)-(3S*, 5S*, 8R*, 9R*, 10S*)-Lab-13-en-3*β*,8*α*-diol-15-oic acid (**8**): white, amorphous solid; [α]_D_^25^ + 5.5 (*c* 0.1, MeOH); IR ν_max_ 3400, 2920, 1700, 1050 cm^−1^; ^1^H NMR (CDCl_3_, 600.13 MHz): *δ*_H_ 5.68 (1H, *bs*, H-14), 3.17 (1H, *dd*, *J* = 4.7, 11.8 Hz, H-3), 2.33 (1H, *td*, *J* = 5.0, 13.2 Hz, H-12a), 2.17 (1H, *td*, *J* = 5.0, 13.2 Hz, H-12b), 2.14 (3H, *s*, H-16), 1.83 (1H, *dt*, *J* = 2.7, 5.4 Hz, H-7a), 1.69 (1H, *m*, H-1a), 1.67 (1H, *m*, H-6a), 1.66 (2H, *m*, H-2), 1.64 (1H, *m*, H-11a), 1.43 (1H, *m*, H-7b), 1.39 (1H, *m*, H-6b), 1.38 (1H, *m*, H-11b), 1.14 (3H, *s*, H-17), 1.13 (1H, *m*, H-1b), 1.09 (1H, *t*, *J* = 3.8, 7.5 Hz, H-9), 0.98 (3H, *s*, H-18), 0.93 (1H, *dd*, *J* = 1.8, 11.8 Hz,H-5), 0.85 (3H, *s*, H-20), 0.76 (3H, *s*, H-19); ^13^C NMR (CDCl_3_, 150.9 MHz): *δ*_C_ 171.0 (C, C-15), 162.0 (C, C-13), 116.9 (CH, C-14), 79.5 (CH, C-3), 74.8 (C, C-8), 62.3 (CH, C-9), 56.5 (CH, C-5), 45.4 (CH_2_, C-12), 45.0 (CH_2_, C-7), 40.1 (C, C-4), 39.9 (CH_2_, C-1), 39.4 (C, C-10), 28.7 (CH_3_, C-18), 27.8 (CH_2_, C-2), 25.1 (CH_2_, C-11), 23.8 (CH_3_, C-17), 21.2 (CH_2_, C-6), 19.1 (CH_3_, C-16), 16.2 (CH_3_, C-20), 16.1 (CH_3_, C-19); HR-ESI-MS *m/z* 337.2391 [M − H]^−^ (calcd. for C_20_H_33_O_4_ 337.2384).

### 3.9. On-flow screening of compounds by immobilized cholinesterase capillary reactors-tandem mass spectrometry—preparation of ICERs

Human recombinant enzyme Hr-AChE (C1682, batch number SLBF4058) and human serum Hu-BChE (B4186, batch number SLBS4001), their substrate acetylcholine (A6625) and standard inhibitor galanthamine (G1660) were purchased from Sigma-Aldrich (St Louis, MO, USA). Hr-AChE-ICER and Hu-BChE-ICER were prepared by covalent enzyme immobilization onto fused silica capillary tubing (30 cm × 0.375 mm × 100 µm I.D.) according to a previously described procedure [22]. Resulting ICERs were connected to an on-flow LC-IT-MS/MS system (EMU FAPESP—Proc. PROEM 2014/50299-5) as biochromatography columns.

#### 3.9.1. LC-IT-MS/MS apparatus

LC system (Shimadzu, Kyoto, Japan) consisted of two Nexera LC-20ADXR pumps, an SIL 20A auto sampler, with a 50 µl loop, a DGU-20A5 degasser and a CBM-20 interface. LC equipment was connected to an AmaZon Speed Ion Trap Mass Spectrometer equipped with an ESI source, operating in positive ion mode. Data were acquired using software Data Analysis 4.1 (Bruker Daltonics). LC analyses were performed at 25°C. Enzymatic reactions were monitored by direct quantification of choline (Ch, [M + H]^+^ *m/z* 104), the hydrolysis product of ACh.

### 3.10. Screening assays

Galanthamine was used as reference inhibitor. Compounds **1** to **8** were tested for Hr-AChE and Hu-BChE inhibition. Samples were solubilized in methanol to provide a stock solution of 1.00 mM for each compound. Assay samples (100 µl) were prepared using 10 µl of stock solution (100 µM final concentration), 20 µl of acetylcholine solution (70 µM final concentration) and 70 µl of ammonium acetate solution (15.0 mM, pH 8.0). Solutions were prepared in duplicate and 20 µl aliquots were used for injection. Negative (absence of ACh) and positive (presence of ACh and absence of ligand) controls were analysed between each AChEI sample. Percentage inhibition displayed by each sample was calculated by comparison between the area of enzymatic activity in the presence of the inhibitor (*P*_i_) and absence (*P*_0_), according to the following equation:

$$\%\mathrm{inhibition}=100-\left[\left(\frac{P_{i}}{P_{0}}\right)\times 100\right]$$

## 4. Conclusion

In this work, the microbial transformation of diterpenoid substrates was performed by two filamentous fungi—*F. oxysporum* and *M. verrucaria*. As results, six oxidized derivatives were obtained, including four new and two known metabolites. Remarkable catalysed-modifications, including oxidations of unactivated C-H sp^3^ bonds, were observed to occur in both rings A and B from substrates into distinct reactivity positions, affording derivatives with further hydroxy and carbonyl functionalities—new reactive sites which can enable accession of a greater number of further analogues. The starting compounds and their biotransformation products were also assayed for anticholinesterase inhibition towards AChE and BChE, through ICER-IT-MS/MS screening. However, only compound **8** showed some enhanced potential over AChE. Therefore, based on the structural modifications from the substrates, it is conclusive that both microorganisms proved to be prolific enzymatic sources for biotransformation of poorly reactive diterpenoids, providing structurally diverse derivatives of valuable chemical and biological relevance.

## Supplementary Material

NMR and MS data of biotransformation products.

## Data accessibility

The datasets supporting this article have been uploaded as electronic supplementary material and are available at the Dryad Digital Repository (https://dx.doi.org/10.5061/dryad.fb7r5) [23].
