# Supplementary material for: Biotransformation of labdane and halimane diterpenoids by two filamentous fungi strains
Source: R Soc Open Sci. 2017 Nov 8;4(11):170854. doi: 10.1098/rsos.170854 (PMC5717651; doi:10.1098/rsos.170854)

**Supplementary Information**

**Biotransformation of labdane and halimane diterpenoids by two filamentous fungi strains**

Afif F. Monteiro,^a^ Cláudia Seidl,^b^ Vanessa G. P. Severino,^c^ Carmen Lúcia Cardoso,^b^ and Ian Castro-Gamboa^*a^

*^a.^ Núcleo de Bioensaios, Biossíntese e Ecofisiologia de Produtos Naturais (NuBBE), Universidade Estadual Paulista (UNESP), Instituto de Química, Departamento de Química Orgânica, Francisco Degni 55, Araraquara, 14800-900, Brazil*

*^b.^ Departamento de Química, Grupo de Cromatografia de Bioafinidade e Produtos Naturais, Faculdade de Filosofia, Ciências e Letras de Ribeirão Preto, Universidade de São Paulo, Ribeirão Preto, 14040-901, SãoPaulo, Brazil*

*^c.^ Universidade Federal de Goiás (UFG), Instituto de Química, Campus Samambaia, Goiânia, 74690-900, Brazil*

* Corresponding author: Tel/Fax: +55 16 3301-9664/3301-9692. E-mail: ian.castro@gmail.com

**Contents**

**S1.** ^1^H NMR (600.13 MHz, CD_3_OD) spectrum of Compound **1**.

**S2.** ^13^C NMR (150.9 MHz, CD_3_OD) spectrum of Compound **1**.

**S3.** ^1^H NMR (600.13 MHz, CD_3_OD) spectrum of Compound **2**.

**S4.** ^13^C NMR (150.9 MHz, CD_3_OD) spectrum of Compound **2**.

**S5.** HRESIMS spectrum of Compound **3** (negative ion mode).

**S6.** ^1^H NMR (600.13 MHz, CD_3_OD) spectrum of Compound **3**.

**S7.** ^13^C NMR (150.9 MHz, CD_3_OD) spectrum of Compound **3**.

**S8.** HSQC (600.13 MHz, CD_3_OD) spectrum of Compound **3**.

**S9.** HMBC (600.13 MHz, CD_3_OD) spectrum of Compound **3**.

**S10.** HRESIMS spectrum of Compound **4** (negative ion mode).

**S11.** ^1^H NMR (600.13 MHz, CDCl_3_) spectrum of Compound **4**.

**S12.** ^13^C NMR (150.9 MHz, CDCl_3_) spectrum of Compound **4**.

**S13.** HSQC (600.13 MHz, CDCl_3_) spectrum of Compound **4**.

**S14.** HMBC (600.13 MHz, CDCl_3_) spectrum of Compound **4**.

**S15.** HRESIMS spectrum of Compound **5** (negative ion mode).

**S16.** ^1^H NMR (600.13 MHz, CDCl_3_) spectrum of Compound **5**.

**S17.** ^13^C NMR (150.9 MHz, CDCl_3_) spectrum of Compound **5**.

**S18.** HSQC (600.13 MHz, CDCl_3_) spectrum of Compound **5**.

**S19.** HMBC (600.13 MHz, CDCl_3_) spectrum of Compound **5**.

**S20.** HRESIMS spectrum of Compound **6** (negative ion mode).

**S21.** ^1^H NMR (600.13 MHz, CDCl_3_) spectrum of Compound **6**.

**S22.** ^13^C NMR (150.9 MHz, CDCl_3_) spectrum of Compound **6**.

**S23.** HSQC (600.13 MHz, CDCl_3_) spectrum of Compound **6**.

**S24.** HMBC (600.13 MHz, CDCl_3_) spectrum of Compound **6**.

**S25.** HRESIMS spectrum of Compound **7** (negative ion mode).

**S26.** ^1^H NMR (600.13 MHz, CD_3_OD) spectrum of Compound **7**.

**S27.** ^13^C NMR (150.9 MHz, CD_3_OD) spectrum of Compound **7**.

**S28.** HSQC (600.13 MHz, CD_3_OD) spectrum of Compound **7**.

**S29.** HMBC (600.13 MHz, CD_3_OD) spectrum of Compound **7**.

**S30.** HRESIMS spectrum of Compound **8** (negative ion mode).

**S31.** ^1^H NMR (600.13 MHz, CD_3_OD) spectrum of Compound **8**.

**S32.** ^13^C NMR (150.9 MHz, CD_3_OD) spectrum of Compound **8**.

**S33.** HSQC (600.13 MHz, CD_3_OD) spectrum of Compound **8**.

**S34.** HMBC (600.13 MHz, CD_3_OD) spectrum of Compound **8**.

**S1.** ^1^H NMR (600.13 MHz, CD_3_OD) spectrum of Compound **1**.


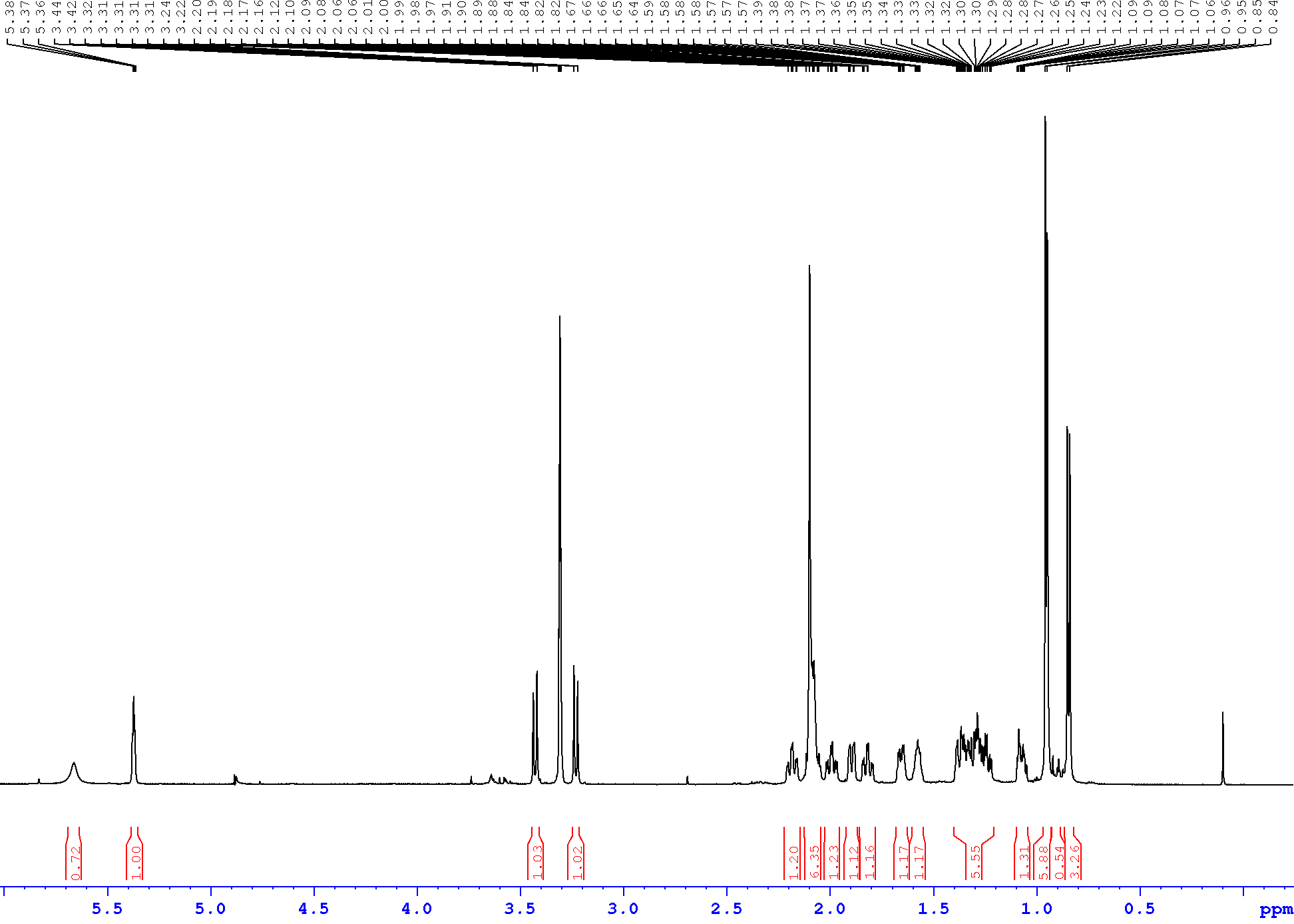


**S2.** ^13^C NMR (150.9 MHz, CD_3_OD) spectrum of Compound **1**.


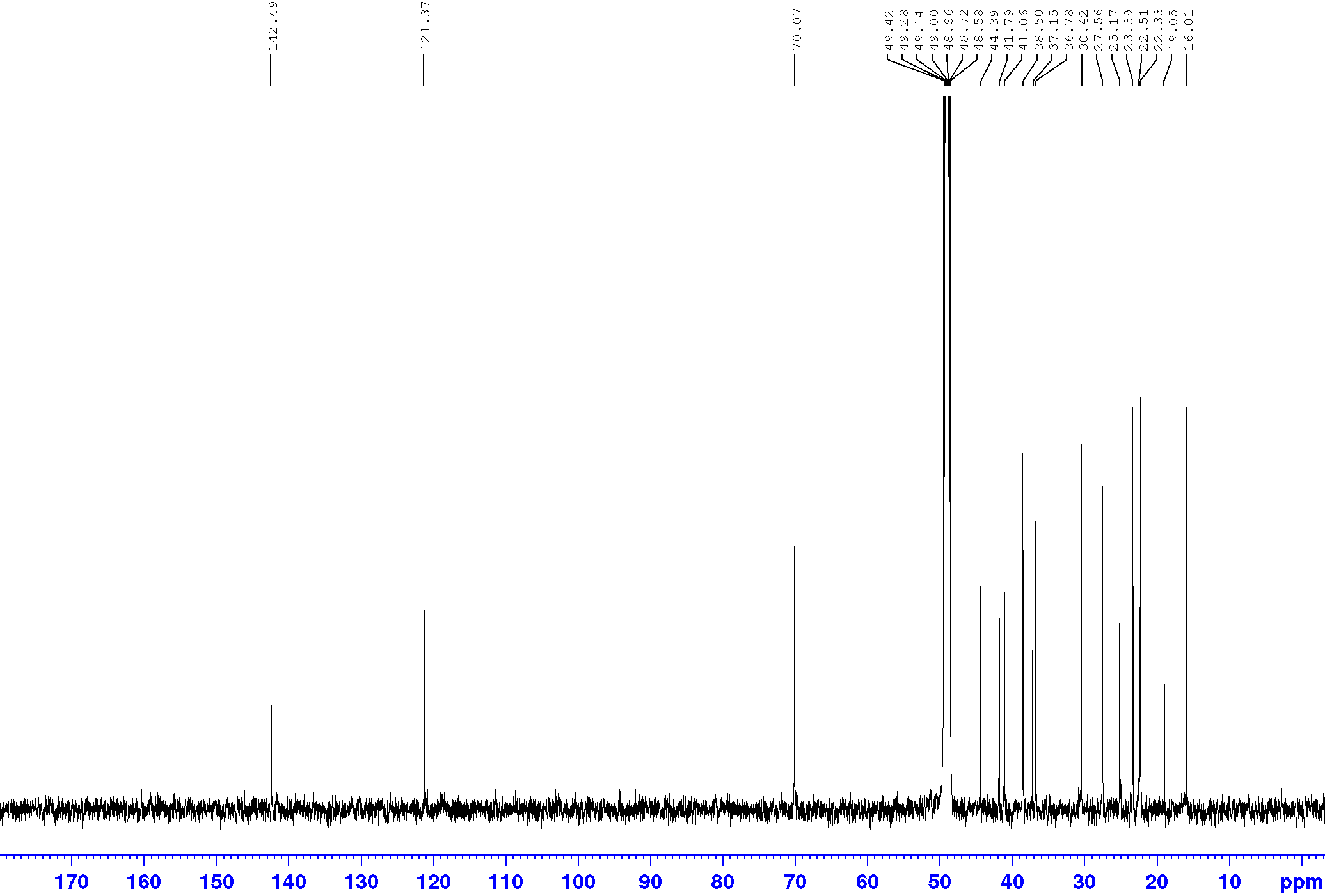


**S3.** ^1^H NMR (600.13 MHz, CD_3_OD) spectrum of Compound **2**.


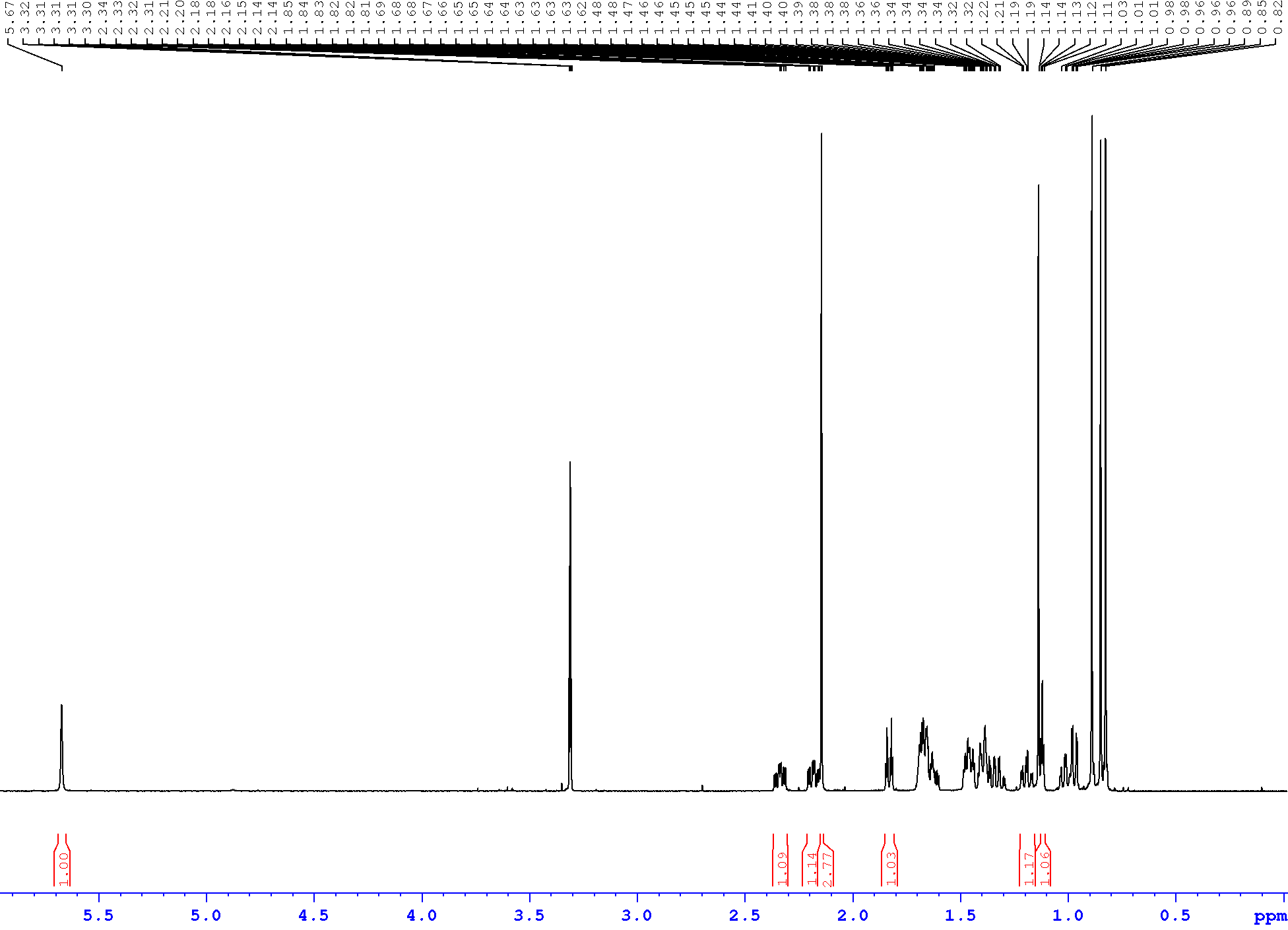


**S4.** ^13^C NMR (150.9 MHz, CD_3_OD) spectrum of Compound **2**.


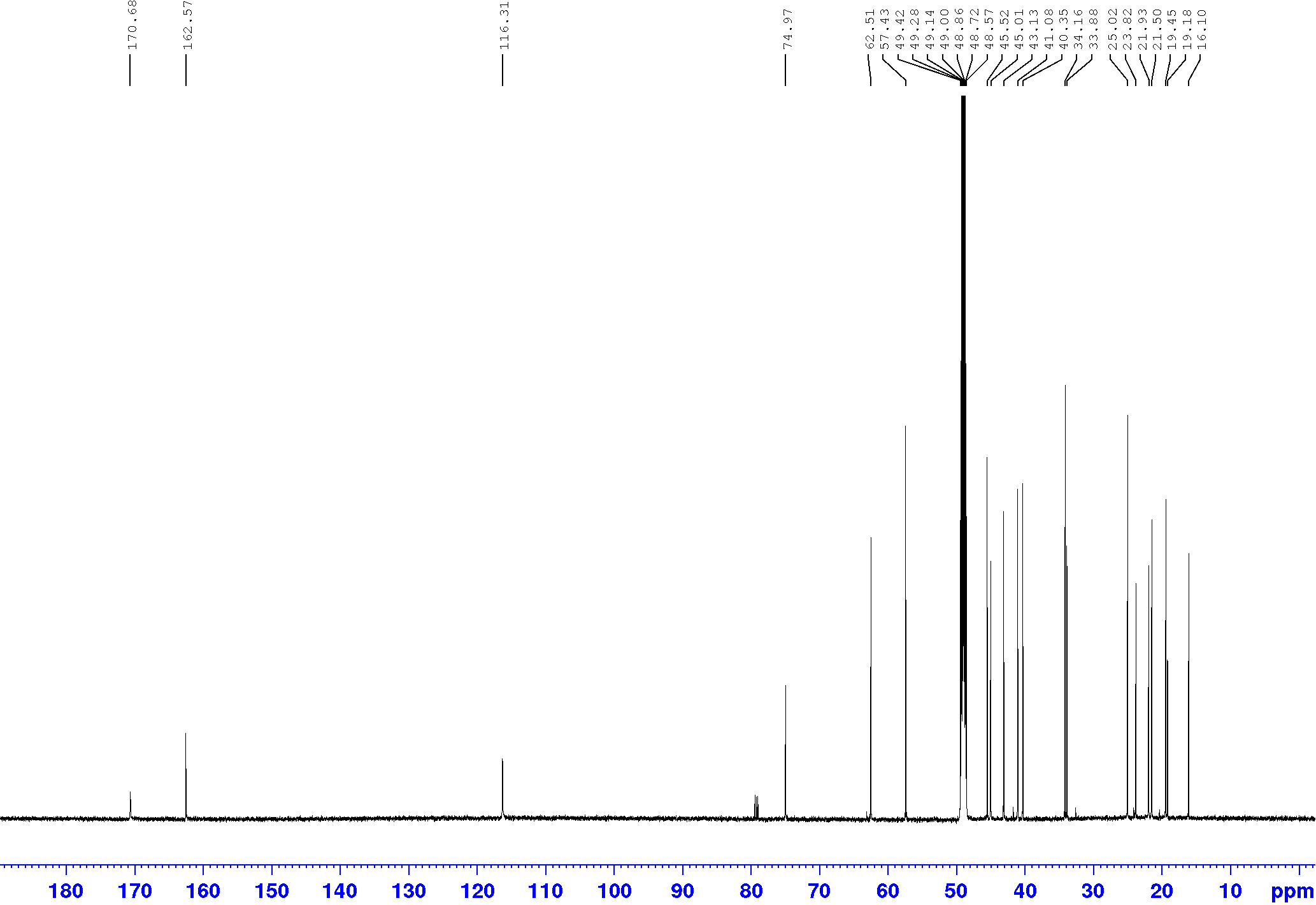


**S5.** HRESIMS spectrum of Compound **3** (negative ion mode).


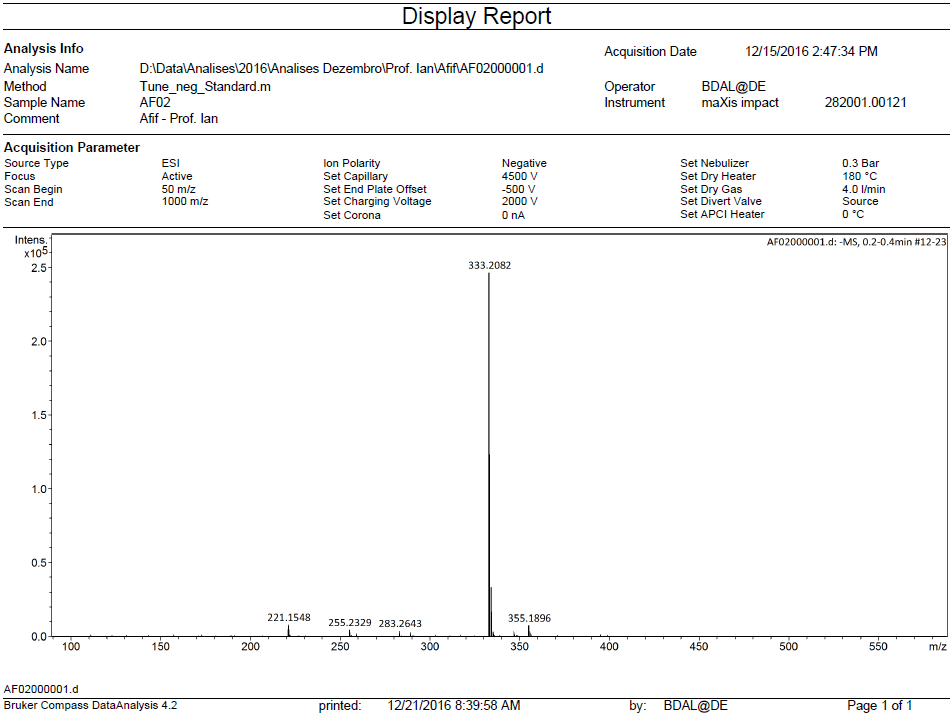


**S6.** ^1^H NMR (600.13 MHz, CD_3_OD) spectrum of Compound **3**.


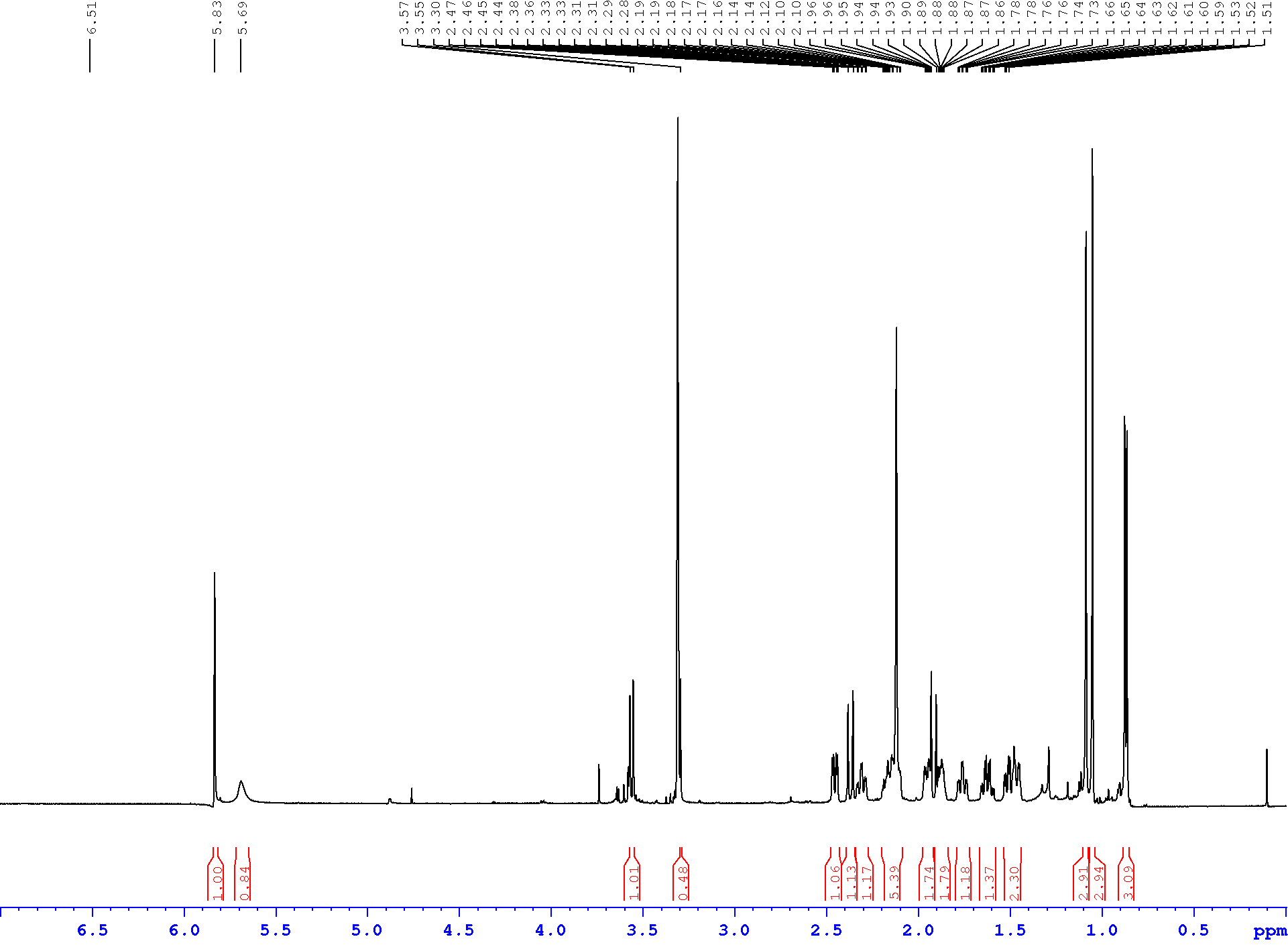


**S7.** ^13^C NMR (150.9 MHz, CD_3_OD) spectrum of Compound **3**.


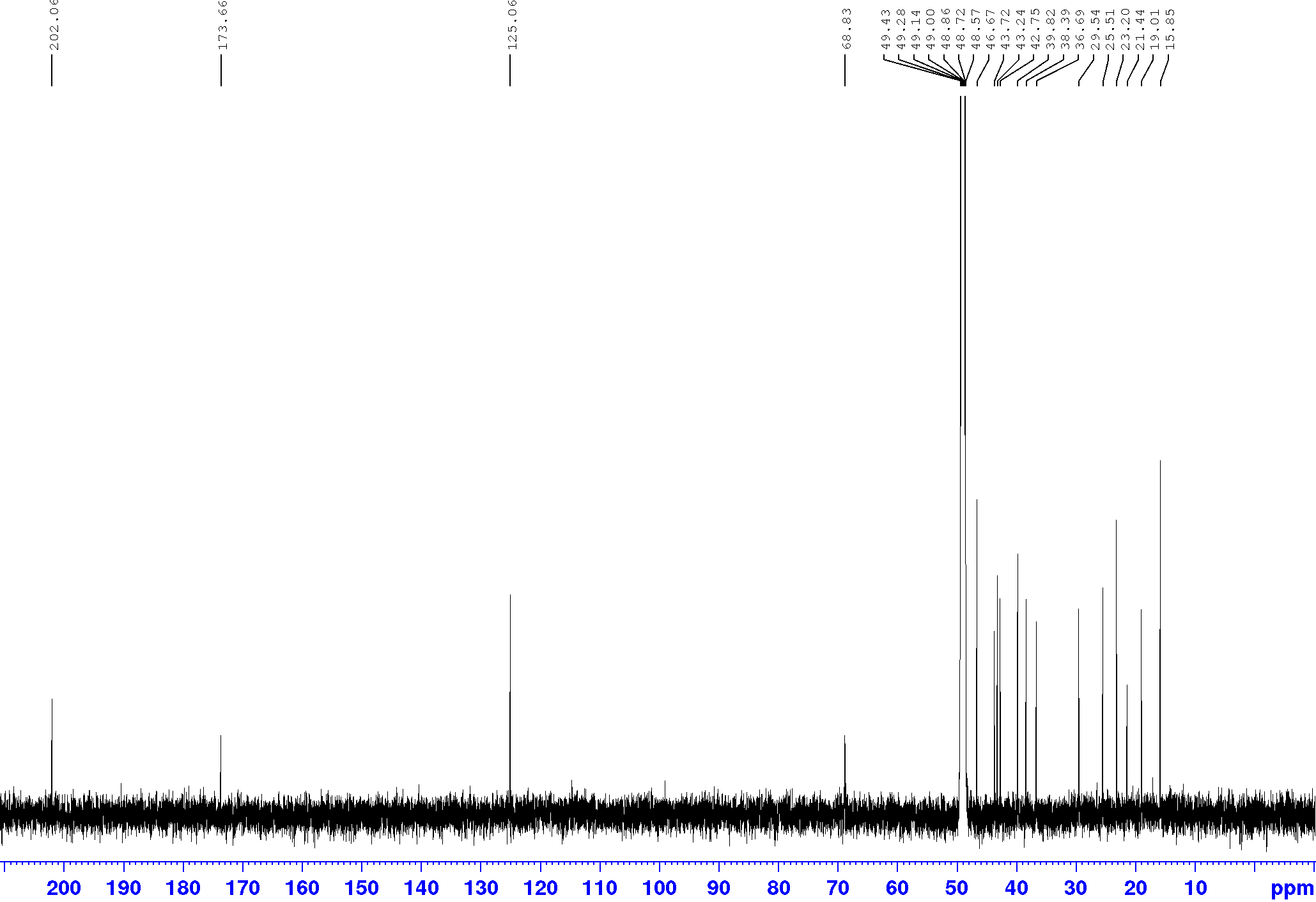


**S8.** HSQC (600.13 MHz, CD_3_OD) spectrum of Compound **3**.


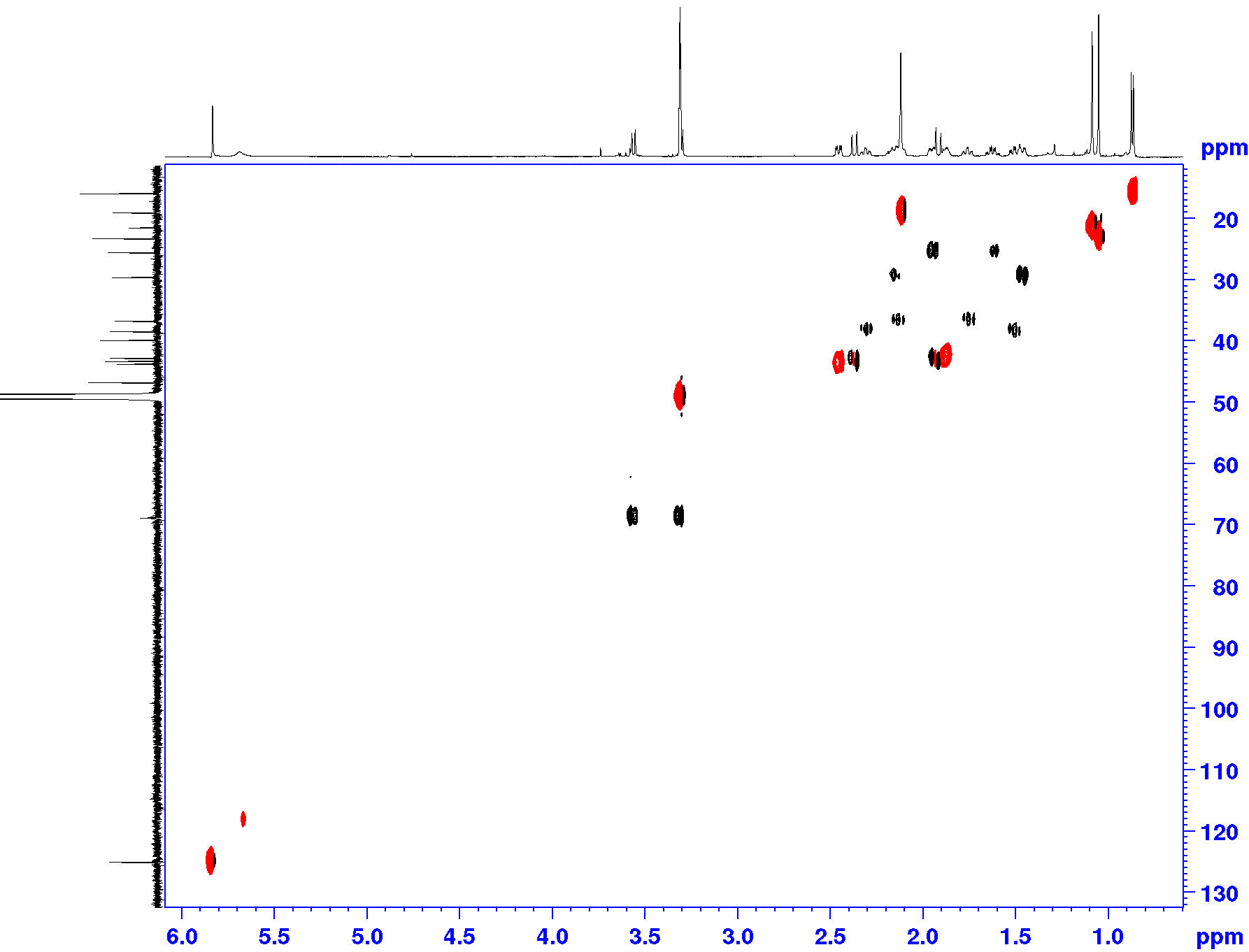


**9.** HMBC (600.13 MHz, CD_3_OD) spectrum of Compound **3**.


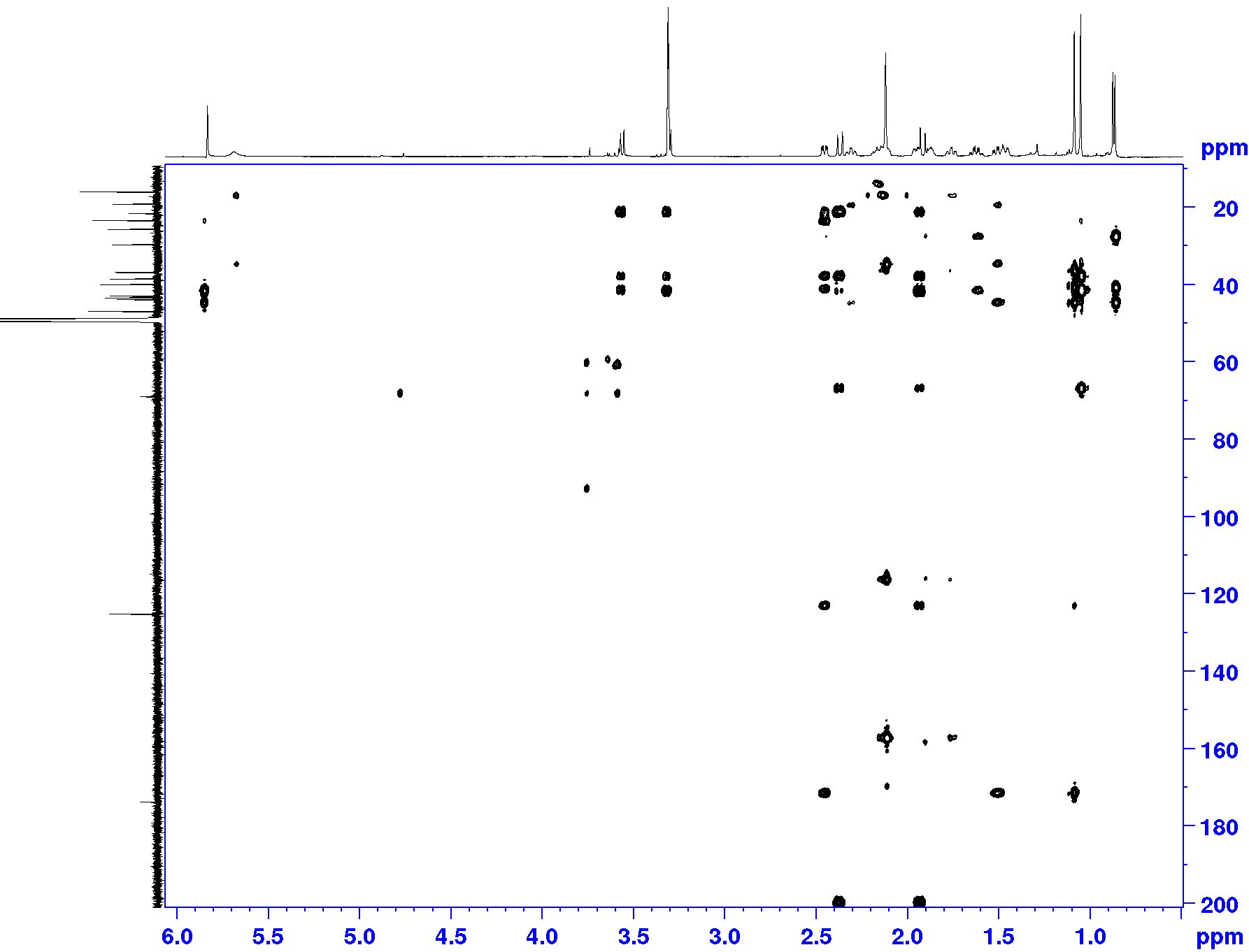


**S10.** HRESIMS spectrum of Compound **4** (negative ion mode).


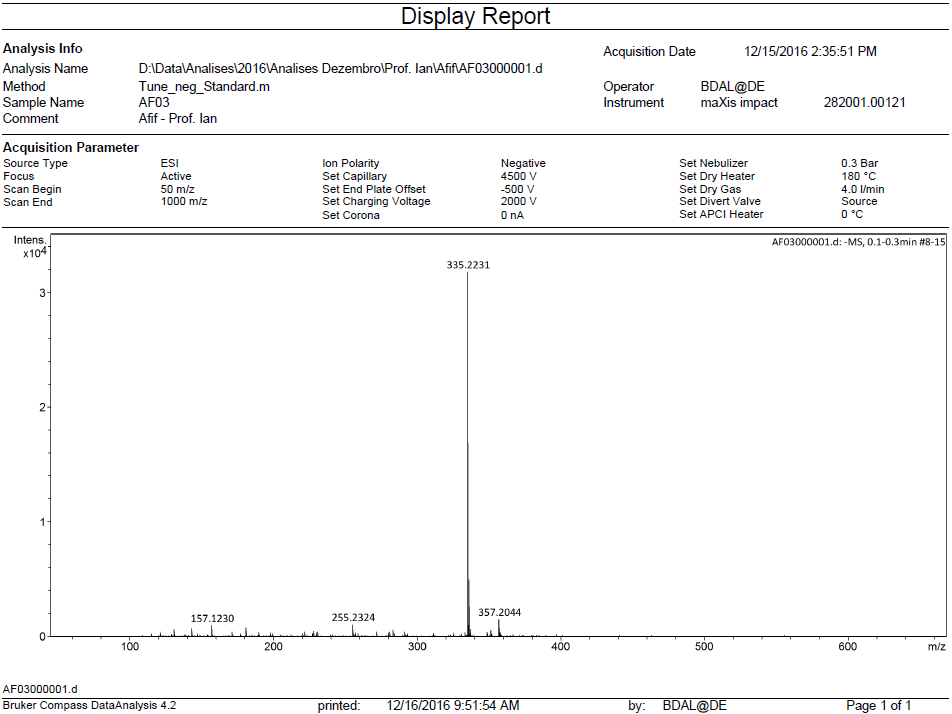


**S11.** ^1^H NMR (600.13 MHz, CDCl_3_) spectrum of Compound **4**.

**
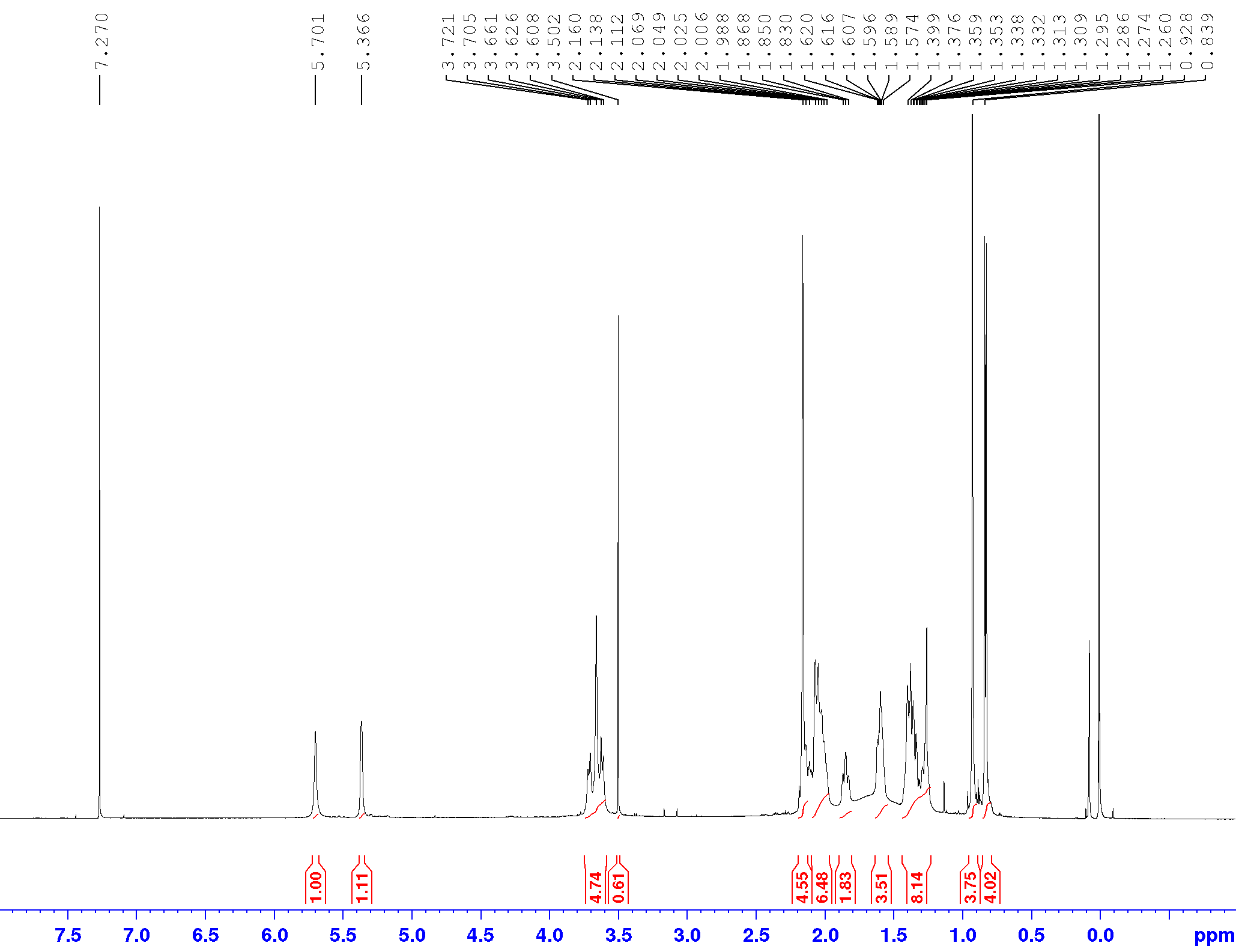
**

**S12.** ^13^C NMR (150.9 MHz, CDCl_3_) spectrum of Compound **4**.


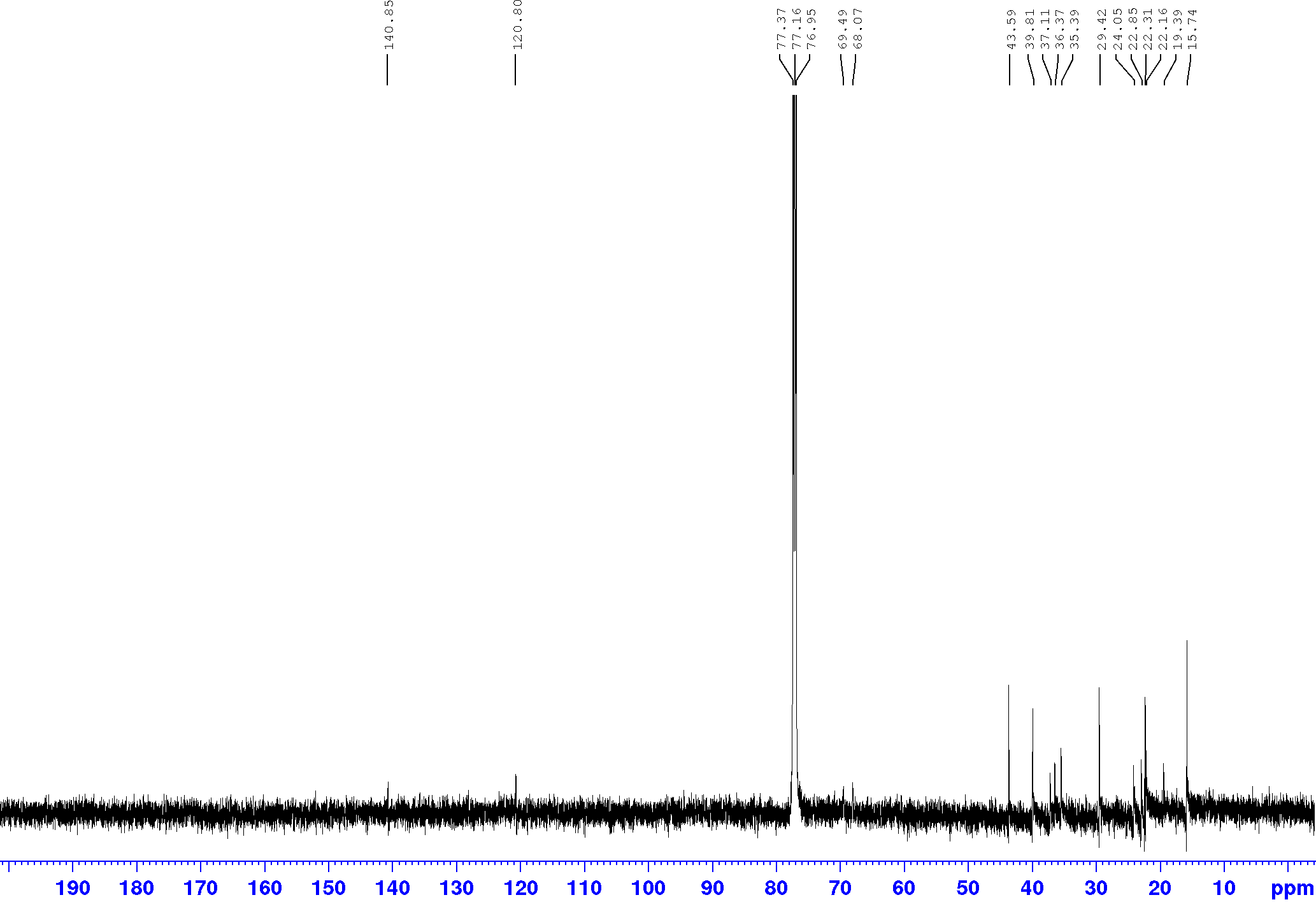


**S13.** HSQC (600.13 MHz, CDCl_3_) spectrum of Compound **4**.


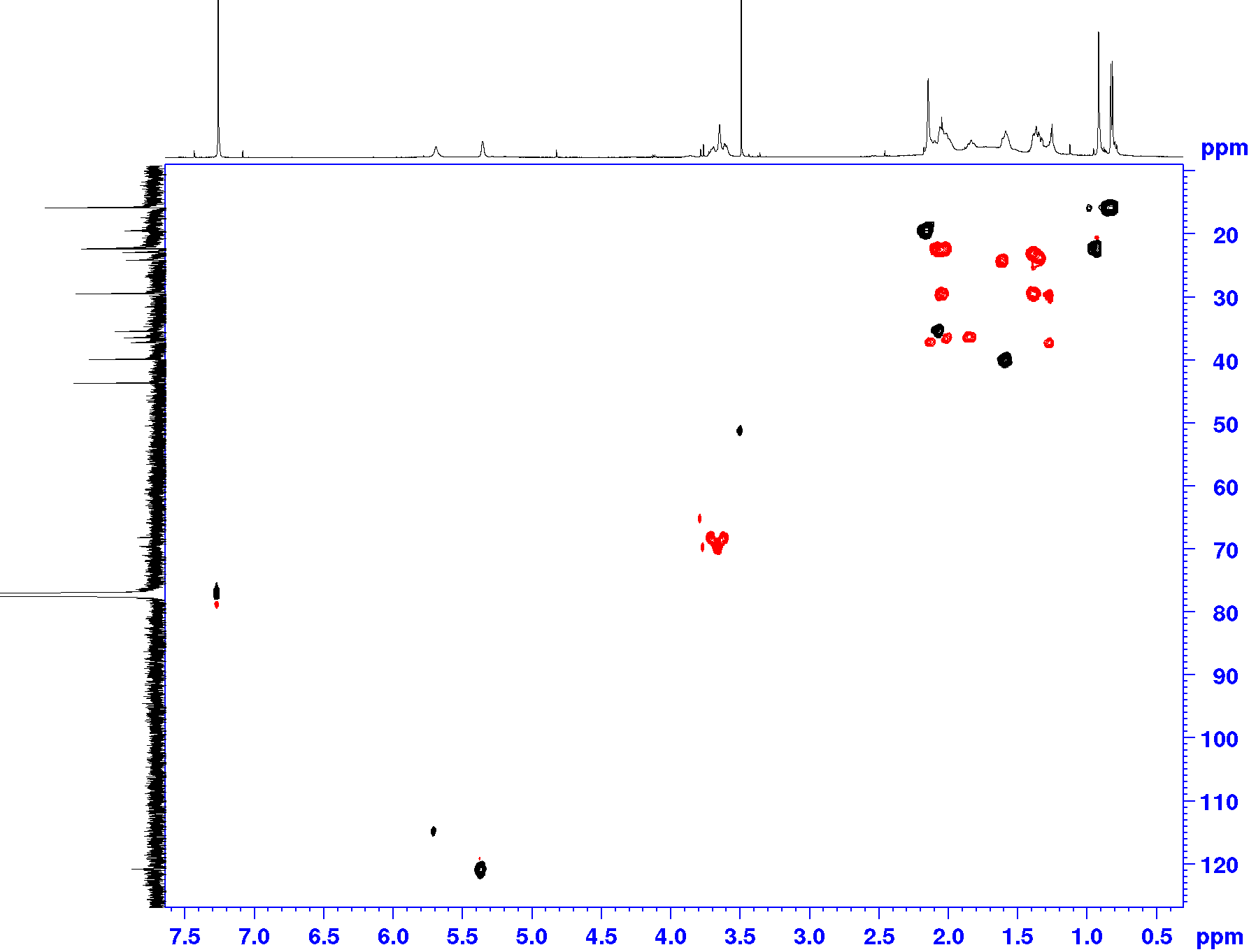


**S14.** HMBC (600.13 MHz, CDCl_3_) spectrum of Compound **4**.


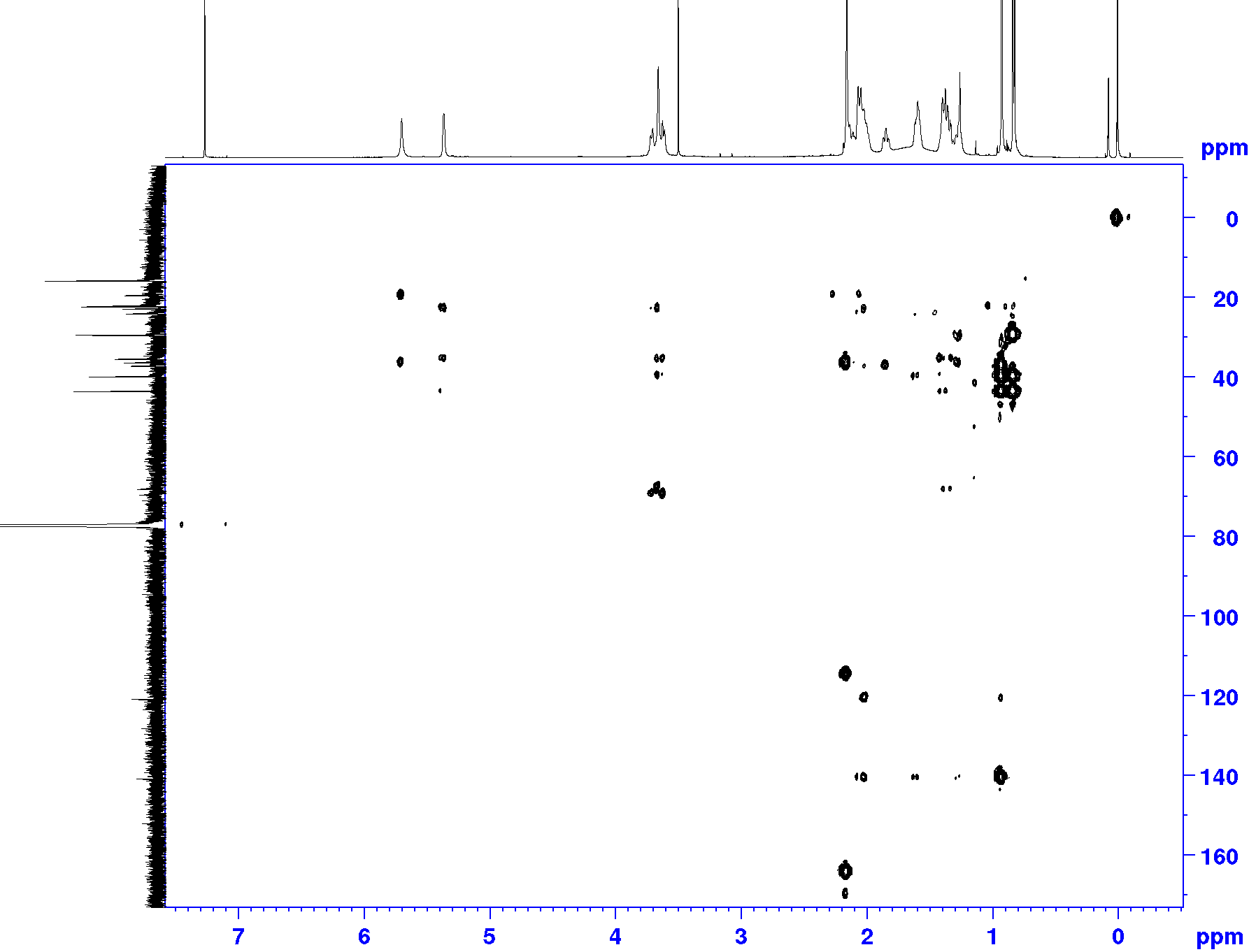


**S15.** HRESIMS spectrum of Compound **5** (negative ion mode).


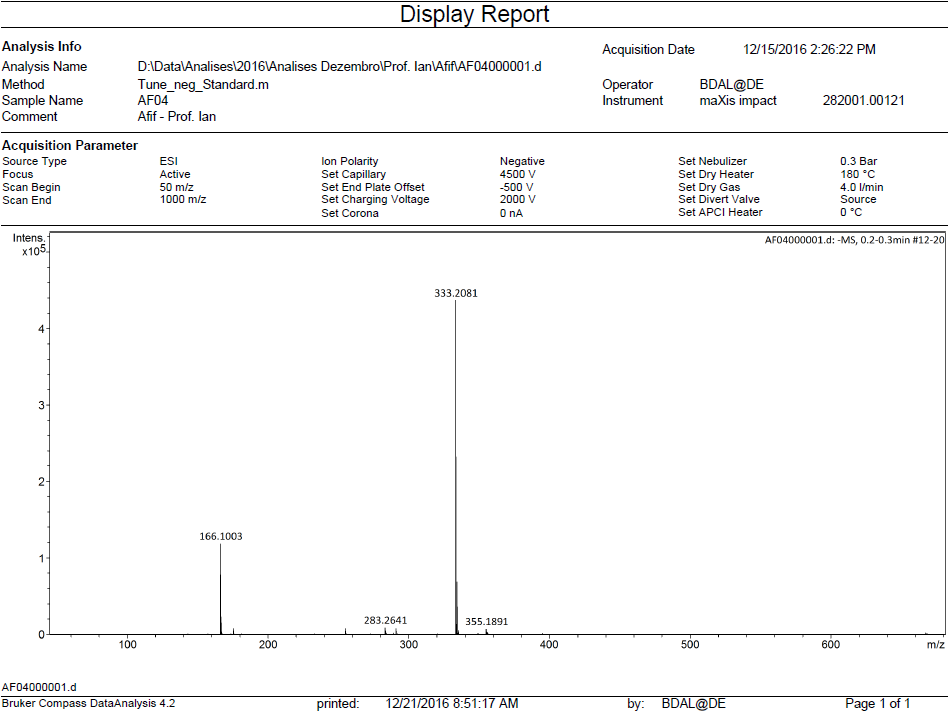


**16.** ^1^H NMR (600.13 MHz, CDCl_3_) spectrum of Compound **5**.


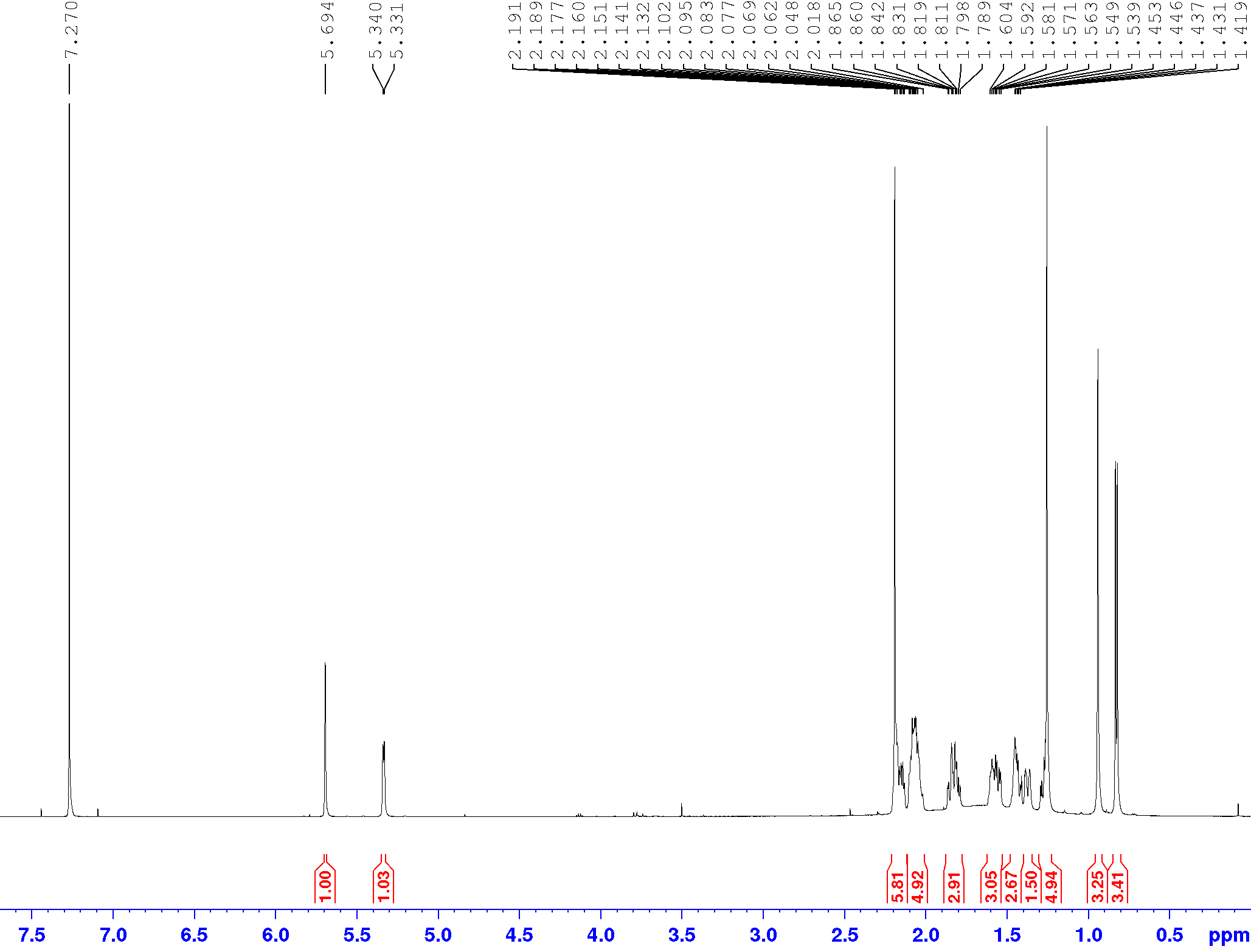


**S17.** ^13^C NMR (150.9 MHz, CDCl_3_) spectrum of Compound **5**.


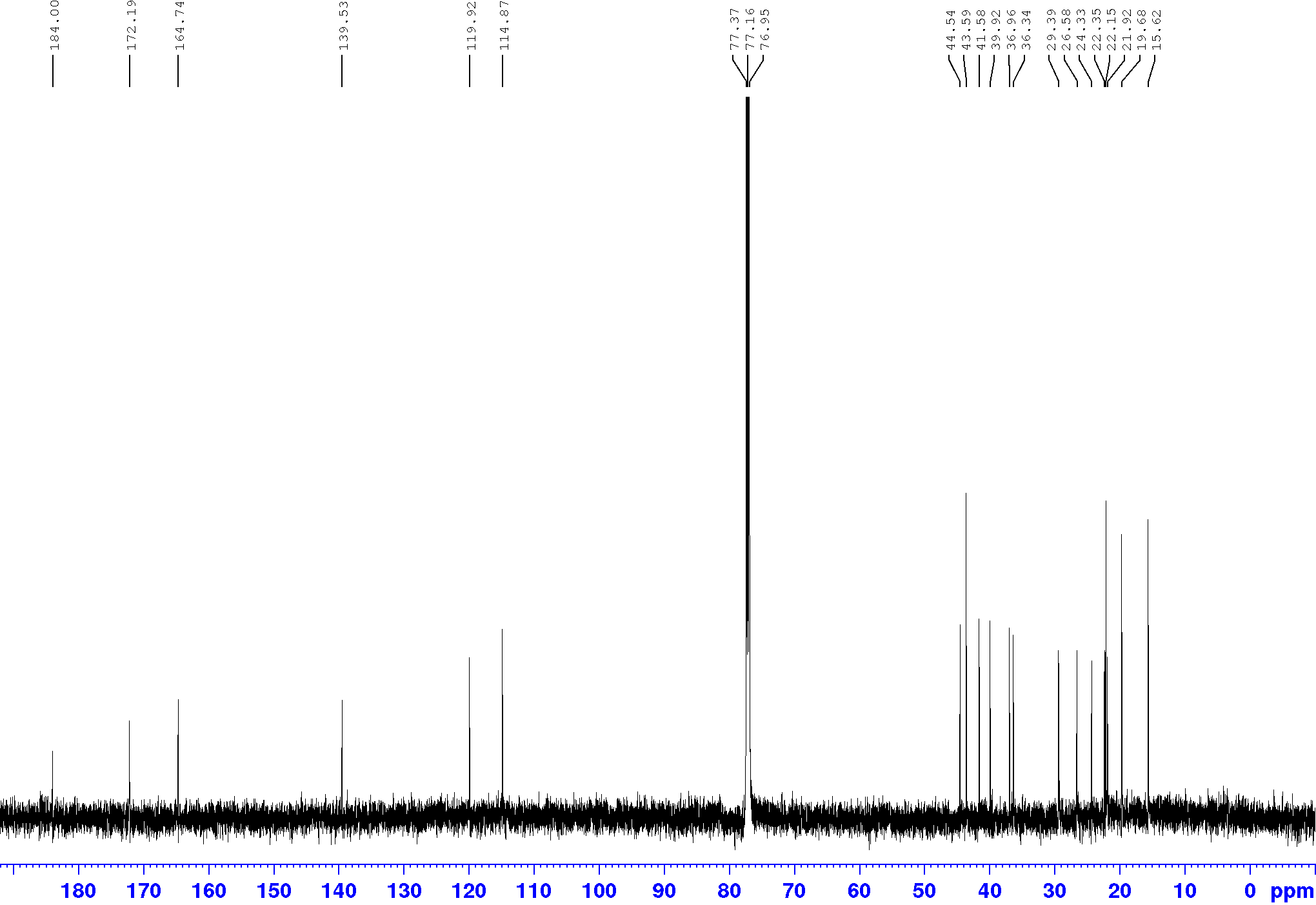


**S18.** HSQC (600.13 MHz, CDCl_3_) spectrum of Compound **5**.


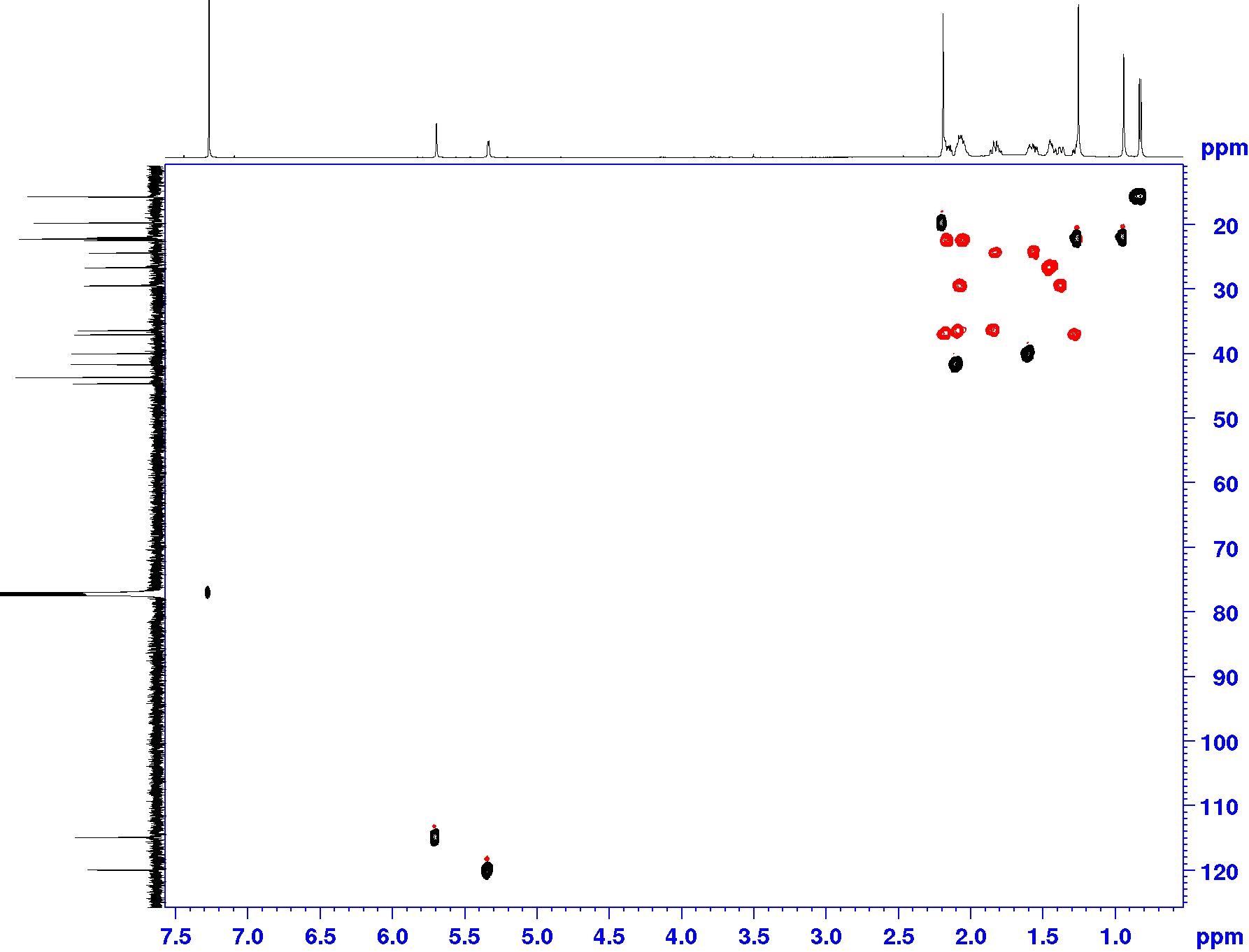


**S19.** HMBC (600.13 MHz, CDCl_3_) spectrum of Compound **5**.


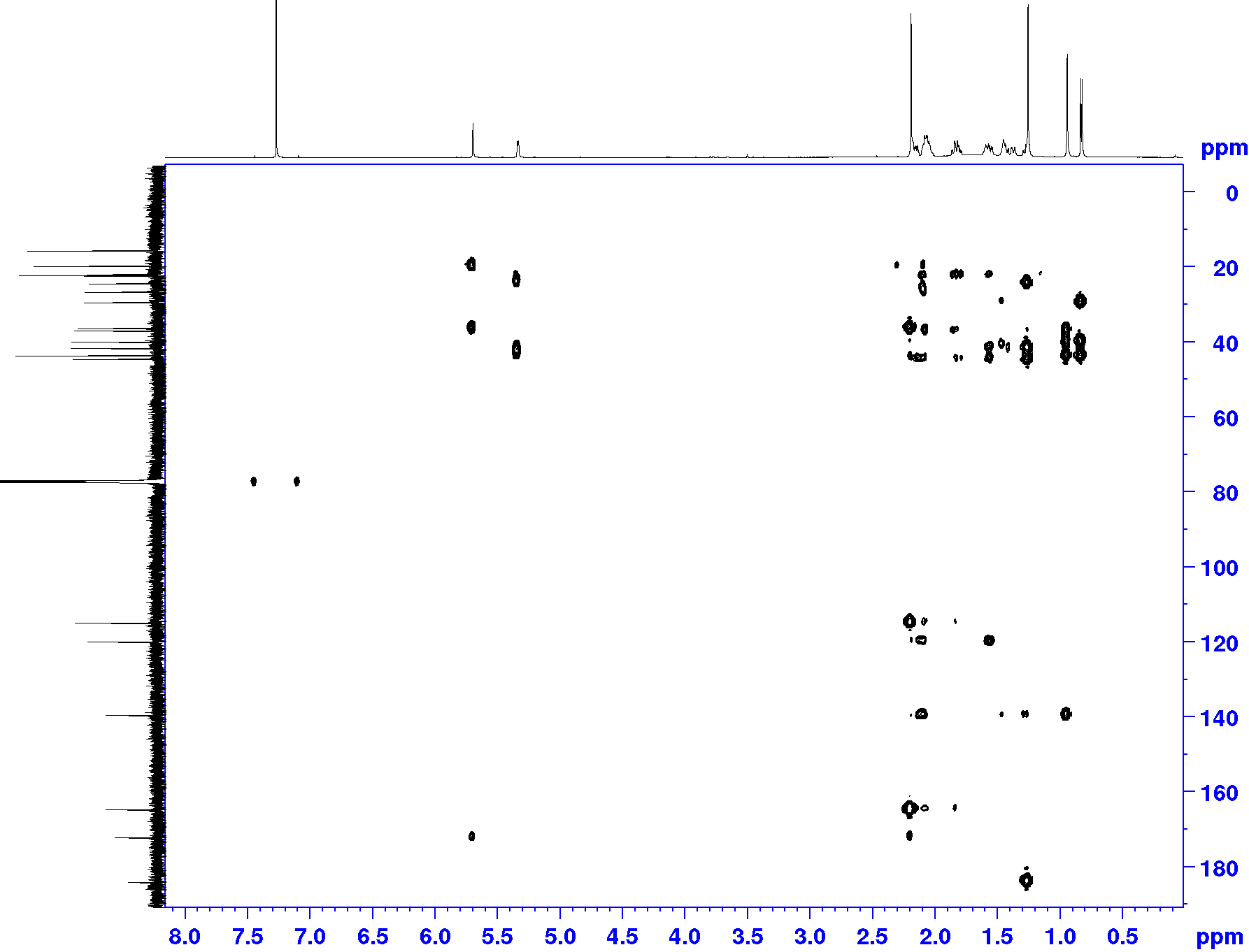


**S20.** HRESIMS spectrum of Compound **6** (negative ion mode).


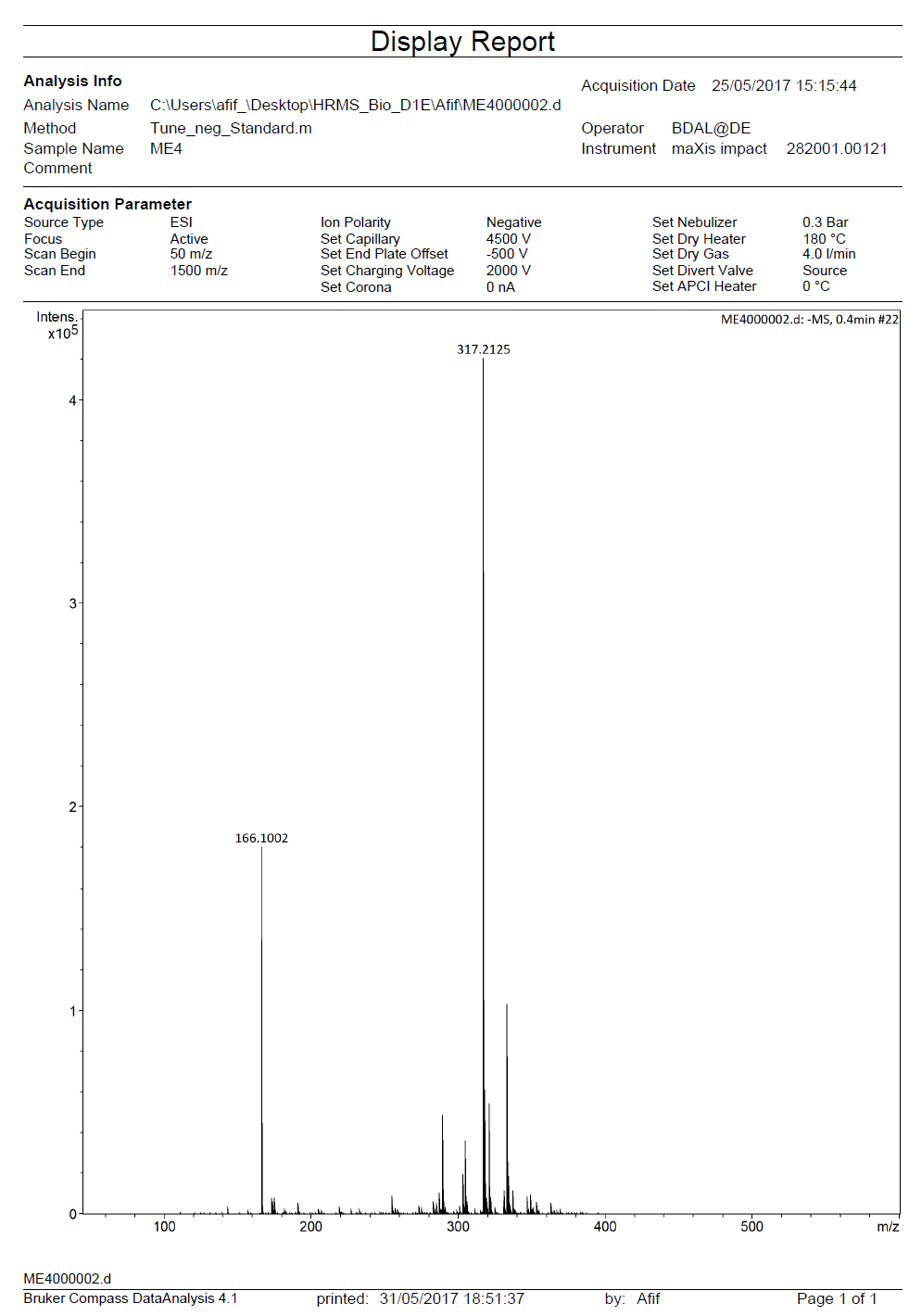


**S21.** ^1^H NMR (600.13 MHz, CDCl_3_) spectrum of Compound **6**.


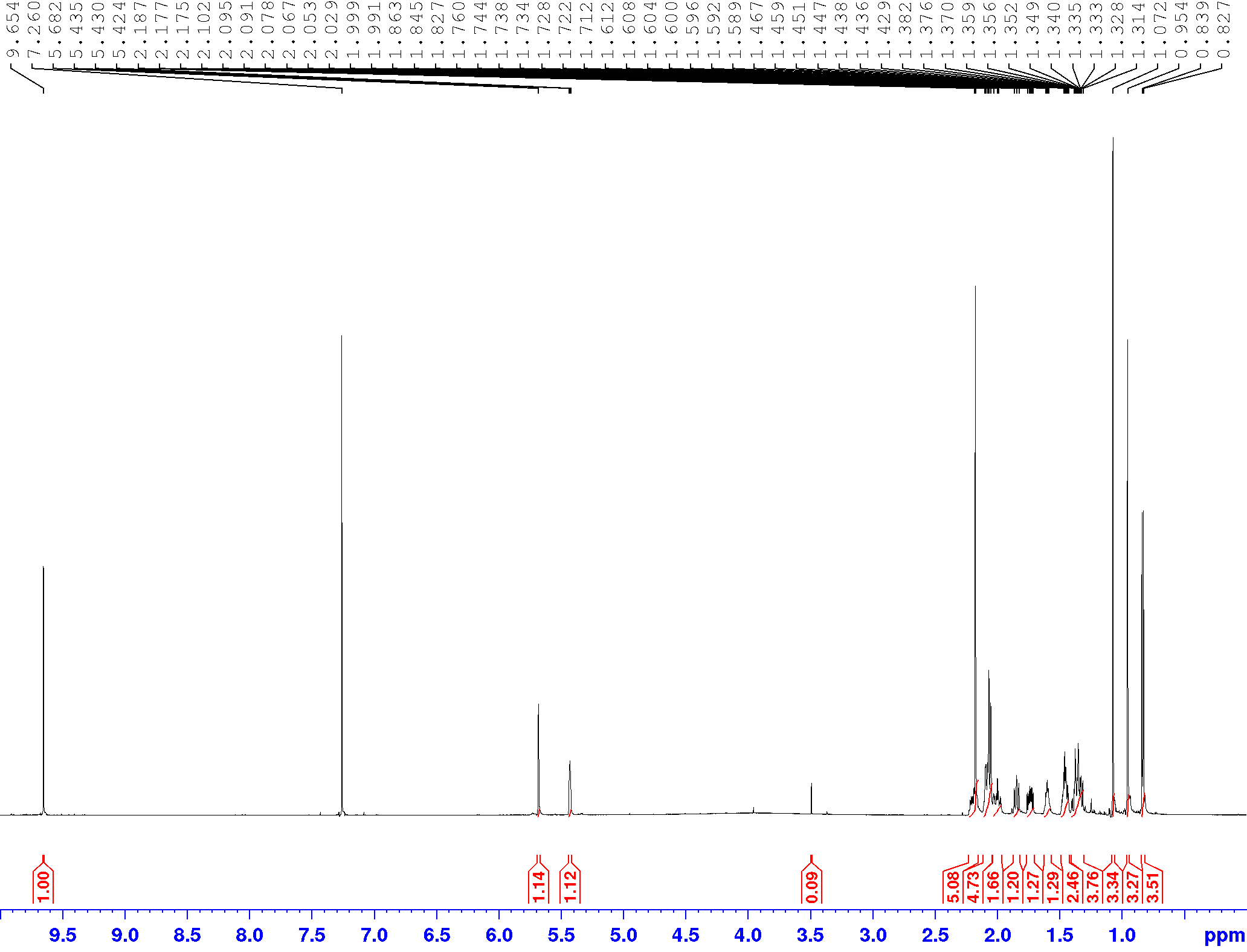


**S22.** ^13^C NMR (150.9 MHz, CDCl_3_) spectrum of Compound **6**.


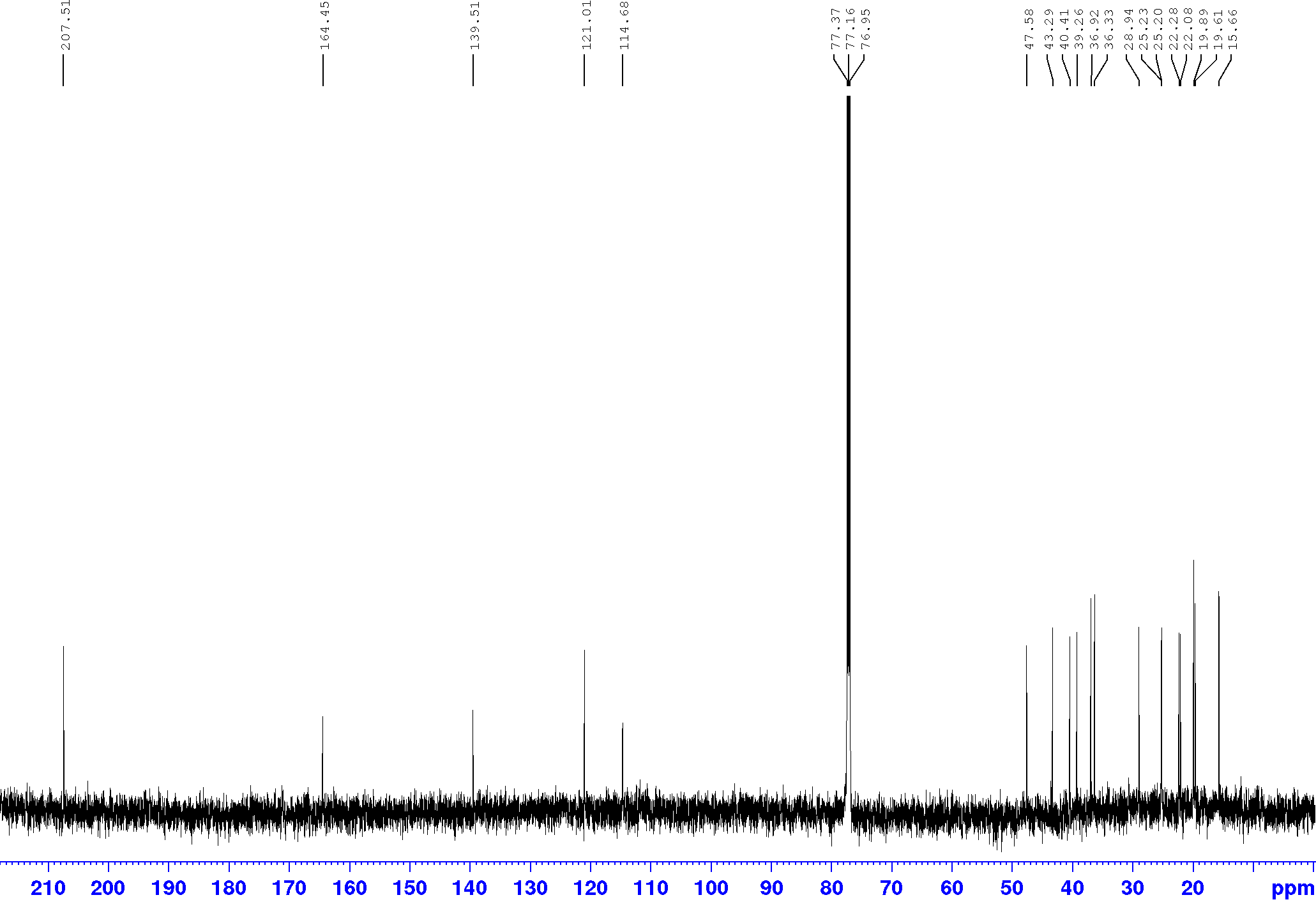


**S23.** HSQC (600.13 MHz, CDCl_3_) spectrum of Compound **6**.


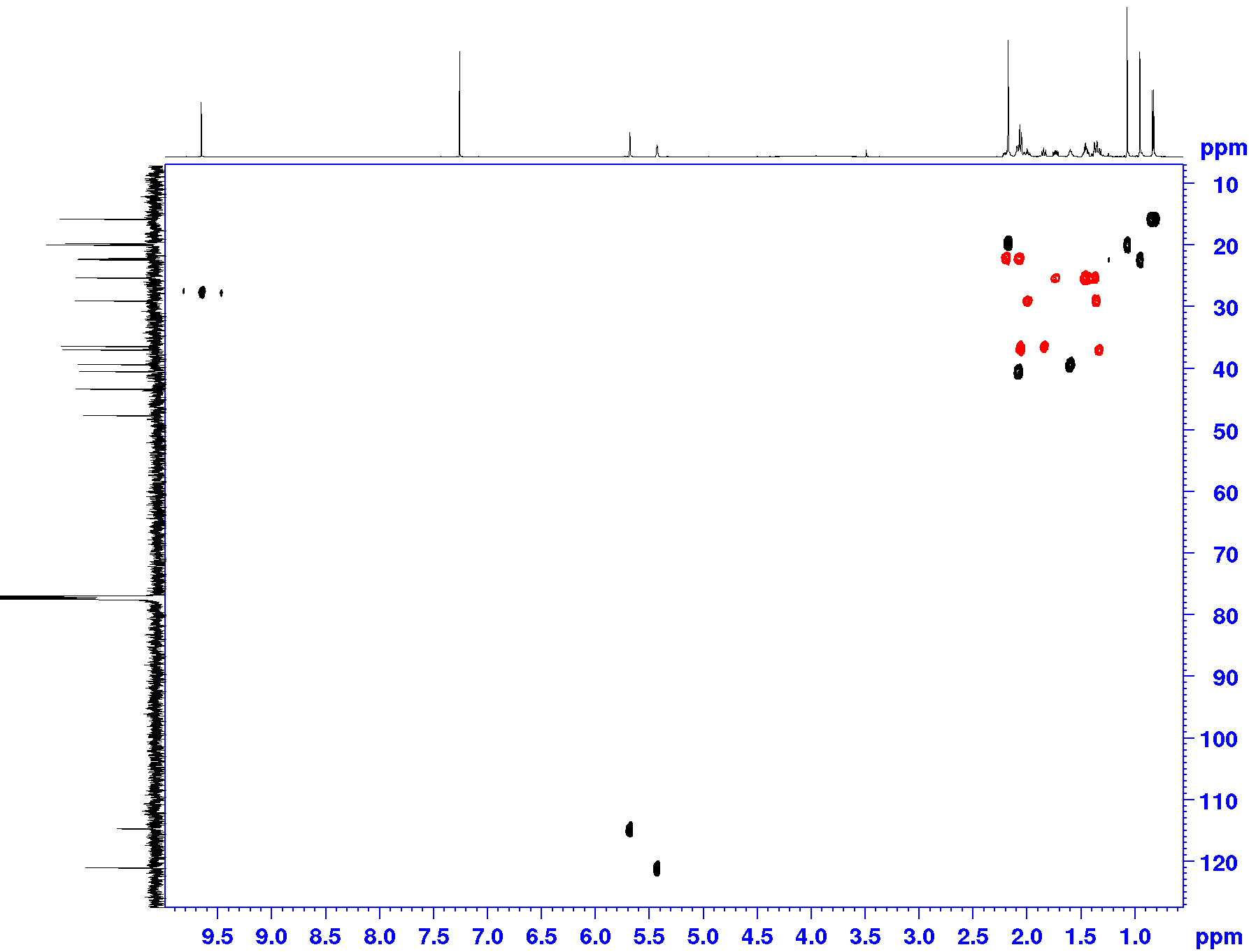


**S24.** HMBC (600.13 MHz, CDCl_3_) spectrum of Compound **6**.


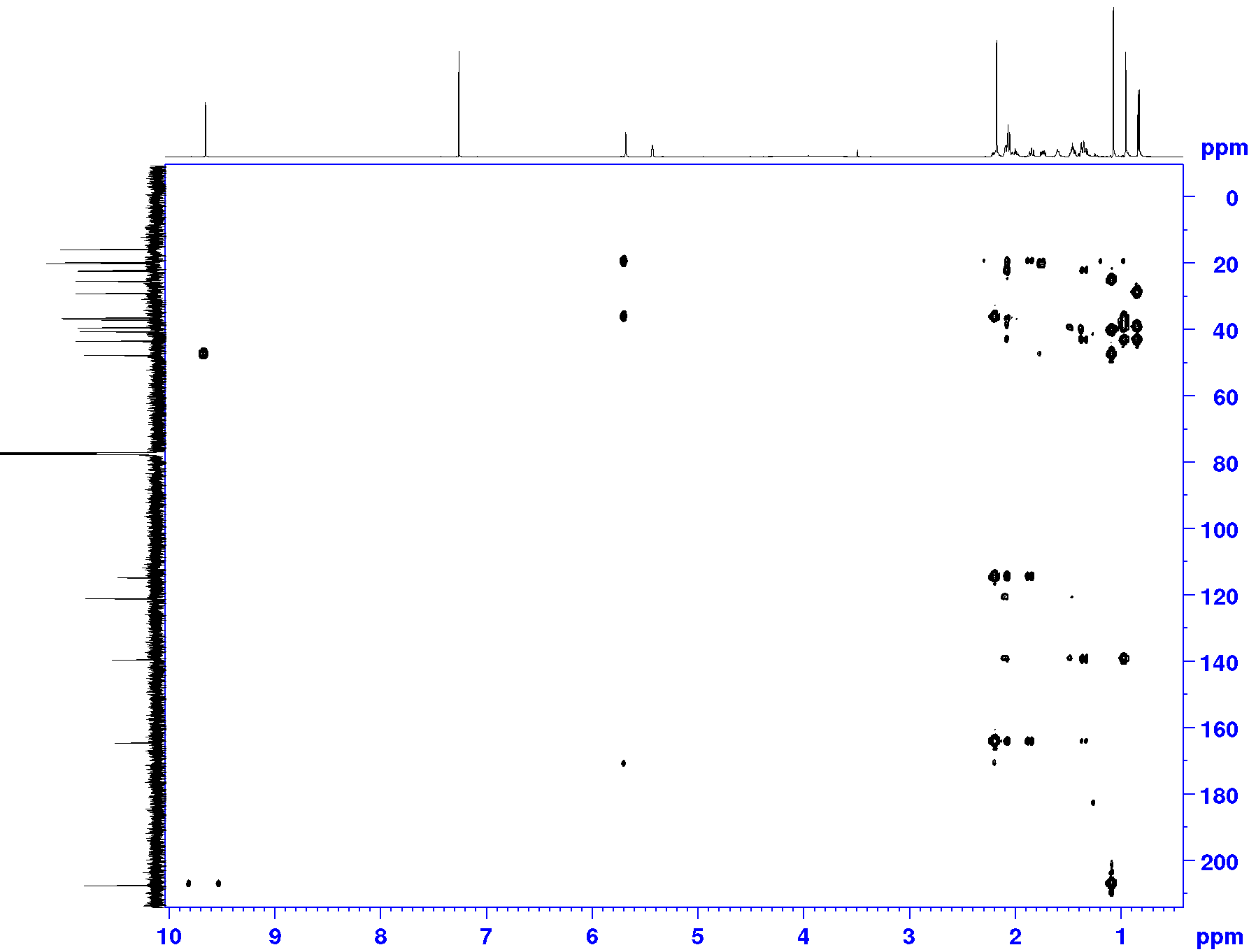


**S25.** HRESIMS spectrum of Compound **7** (negative ion mode).


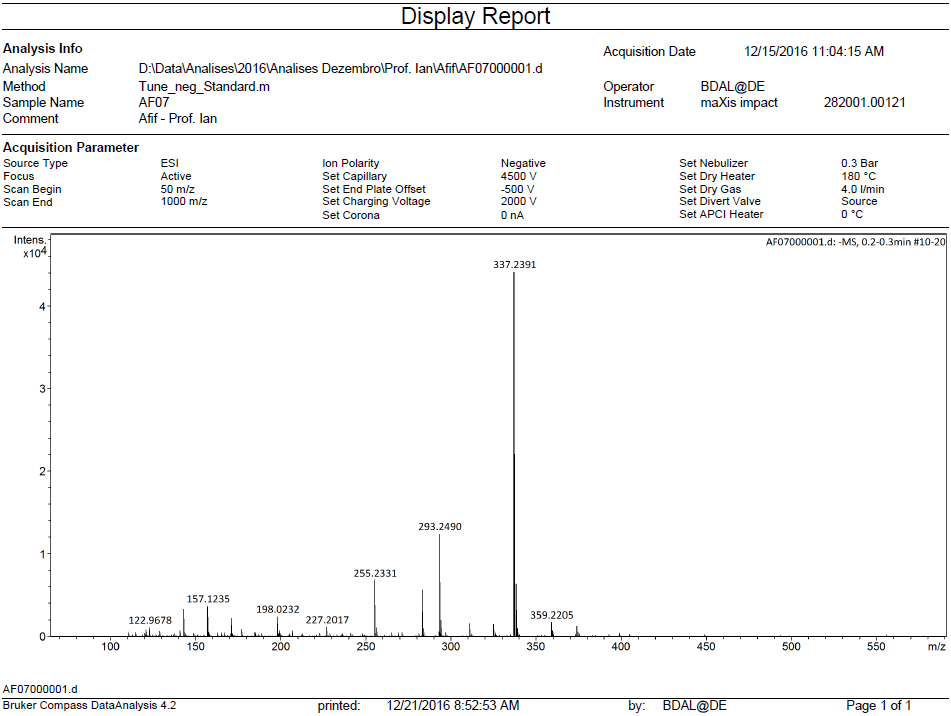


**S26.** ^1^H NMR (600.13 MHz, CD_3_OD) spectrum of Compound **7**.


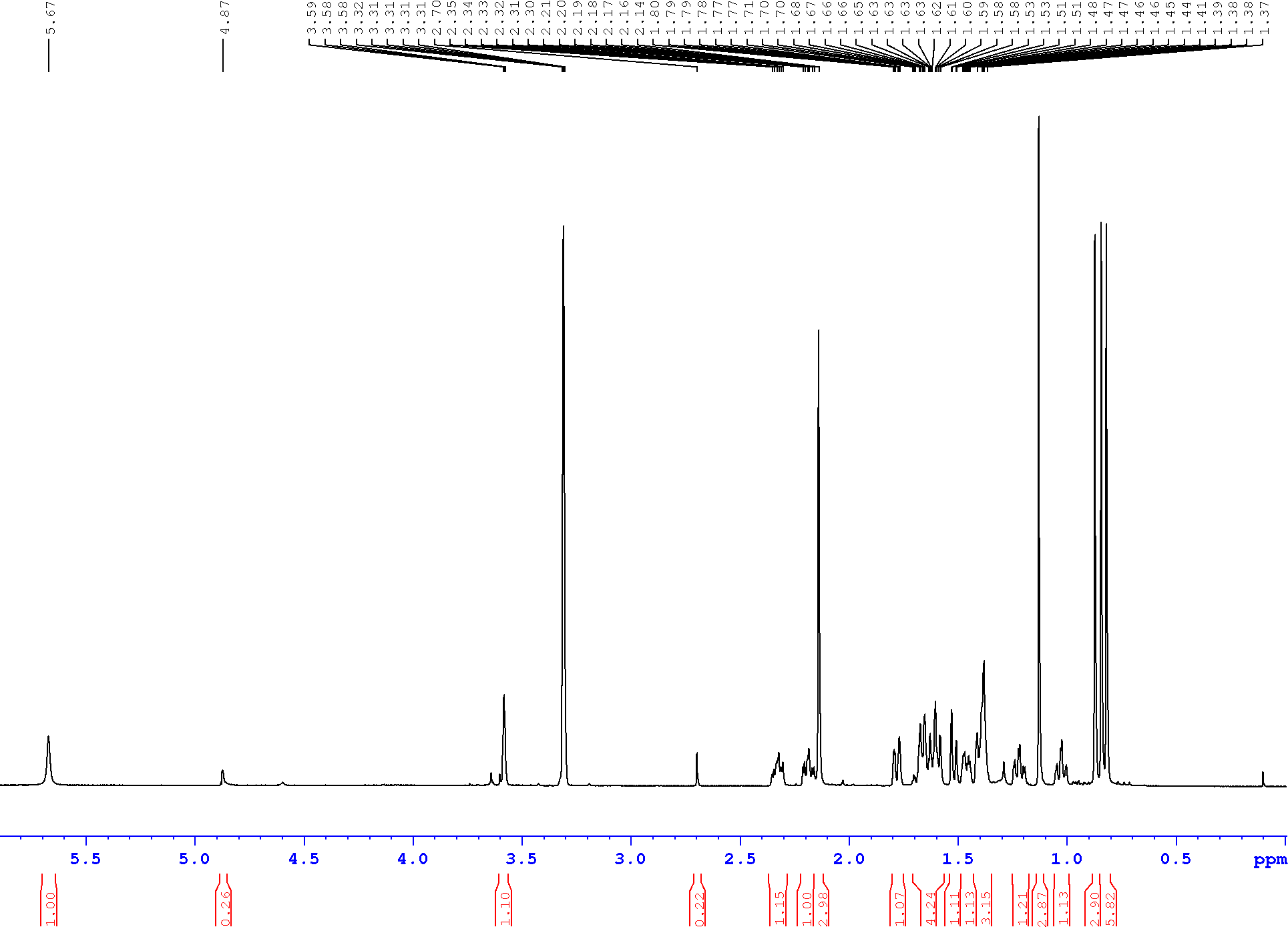


**S27.** ^13^C NMR (150.9 MHz, CD_3_OD) spectrum of Compound **7**.


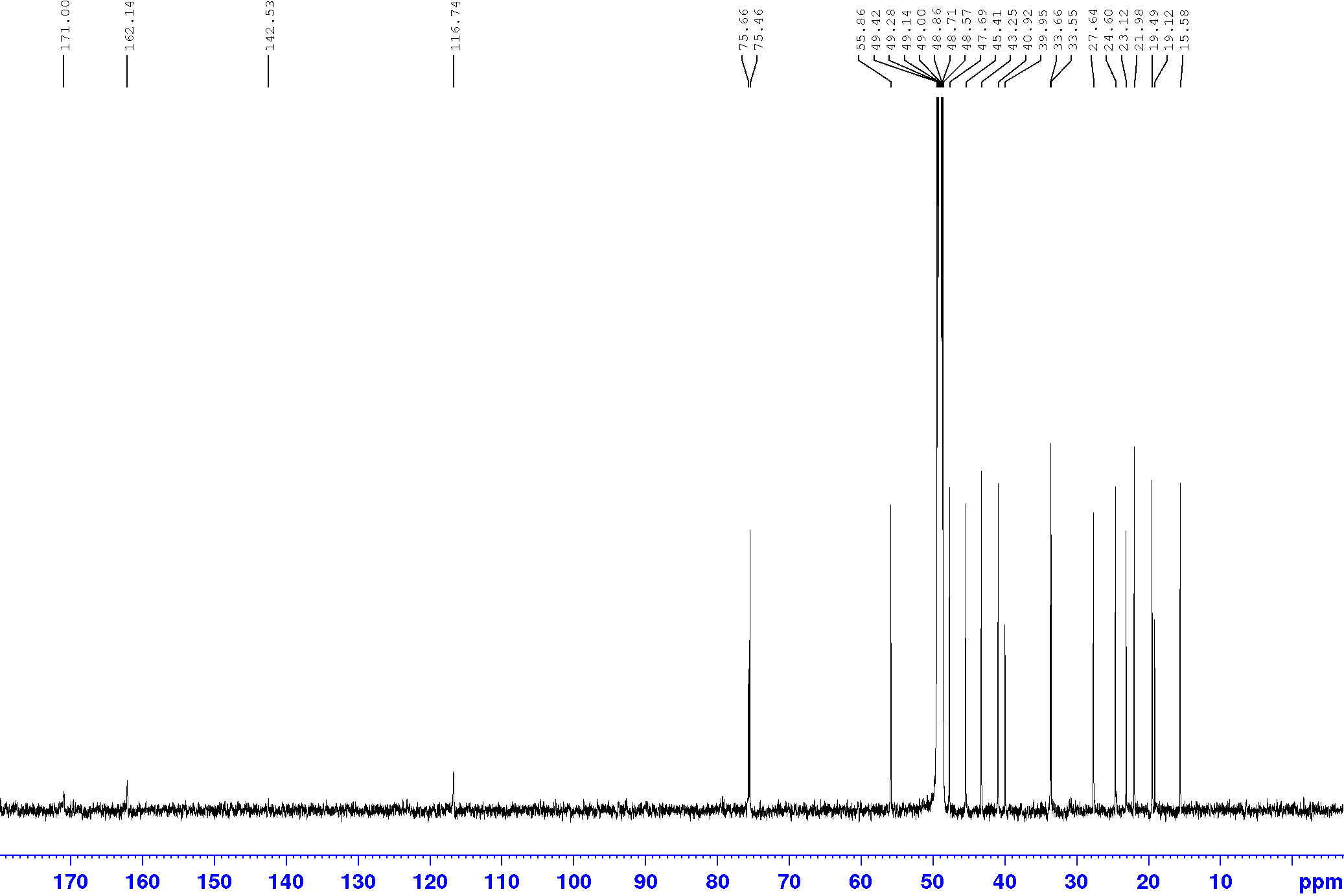


**S28.** HSQC (600.13 MHz, CD_3_OD) spectrum of Compound **7**.

**
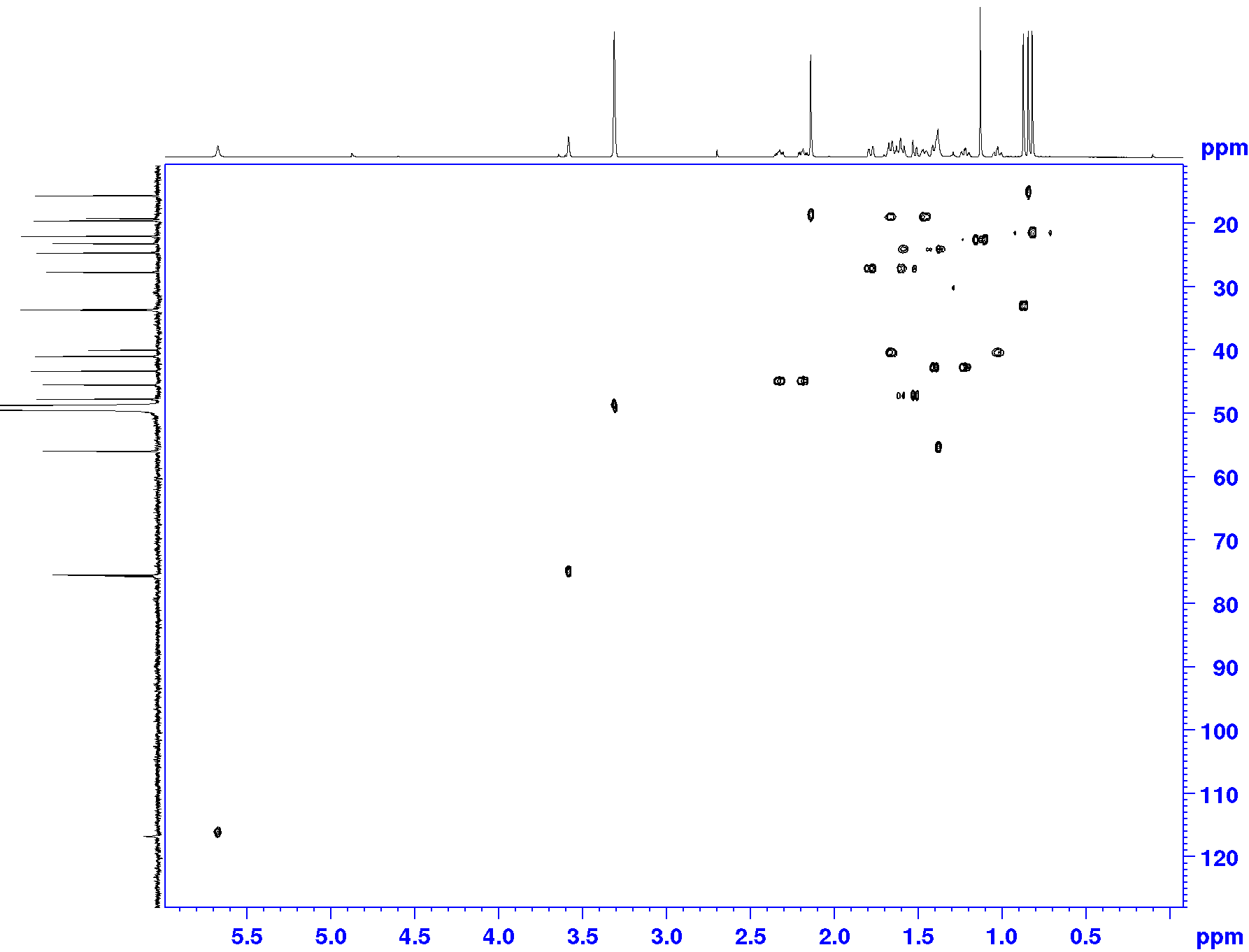
**

**S29.** HMBC (600.13 MHz, CD_3_OD) spectrum of Compound **7**.


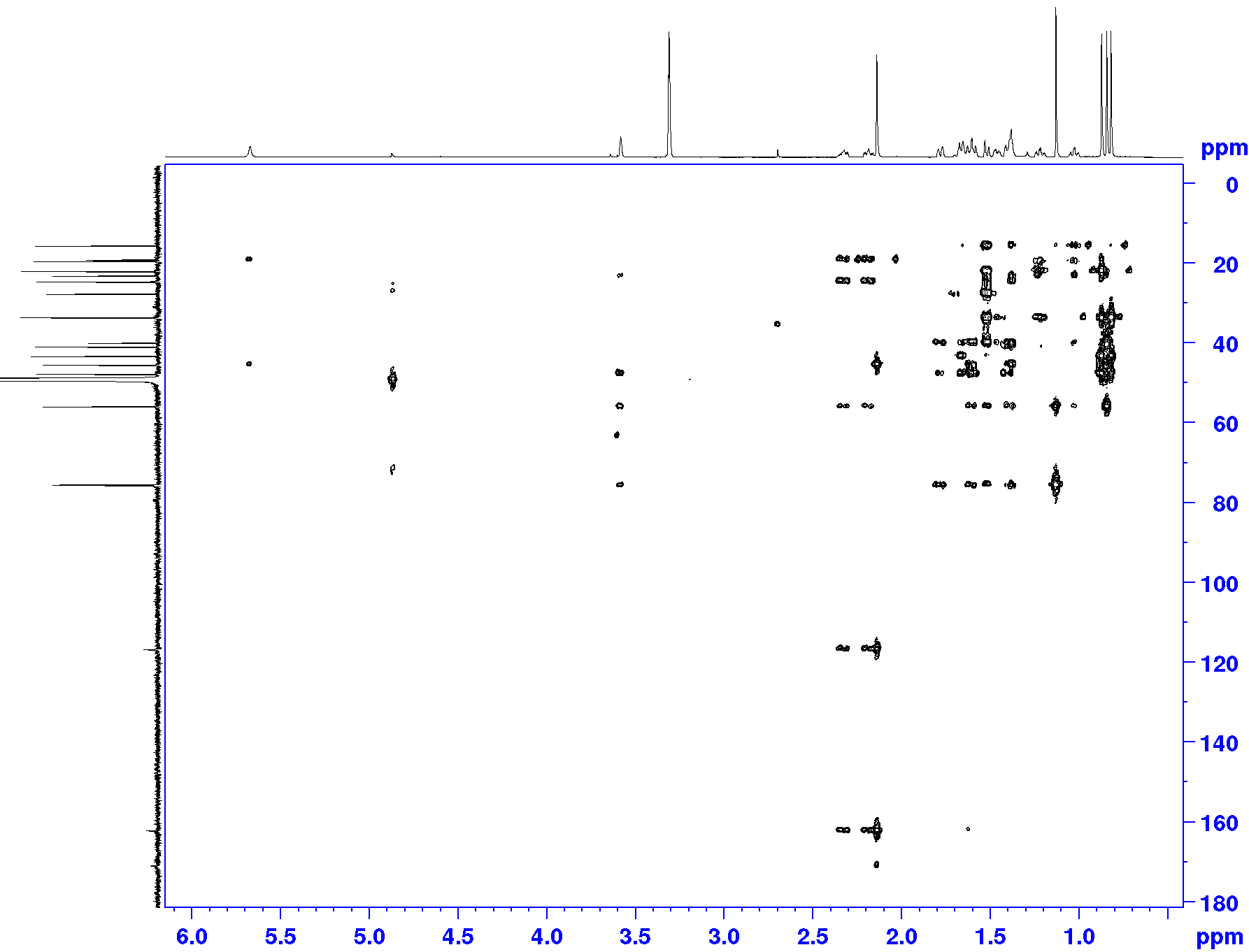


**S30.** HRESIMS spectrum of Compound **8** (negative ion mode).


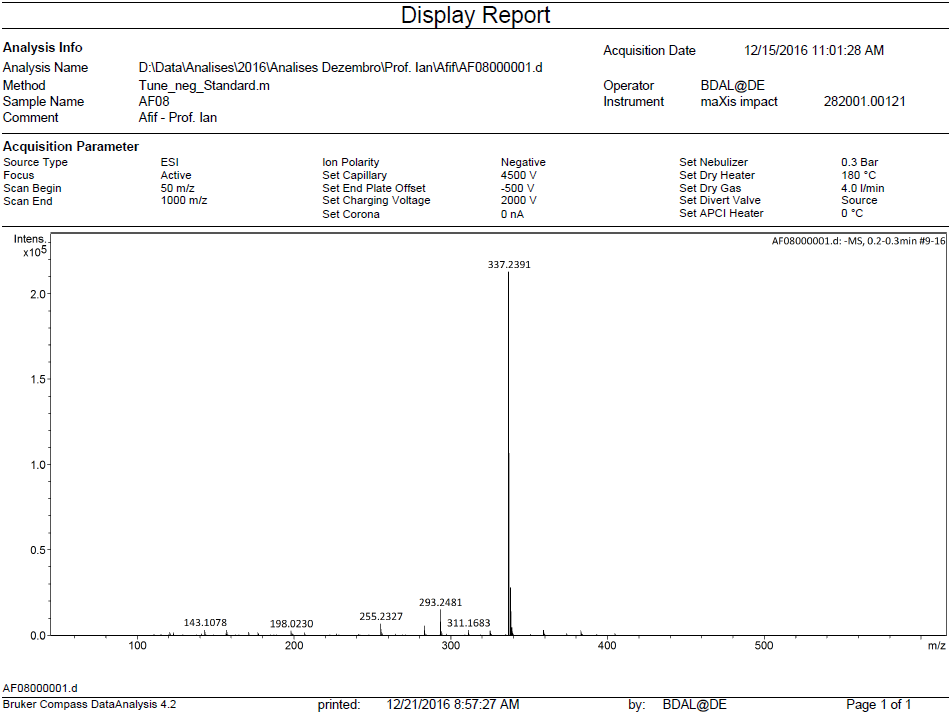


**S31.** ^1^H NMR (600.13 MHz, CDCl_3_) spectrum of Compound **8**.


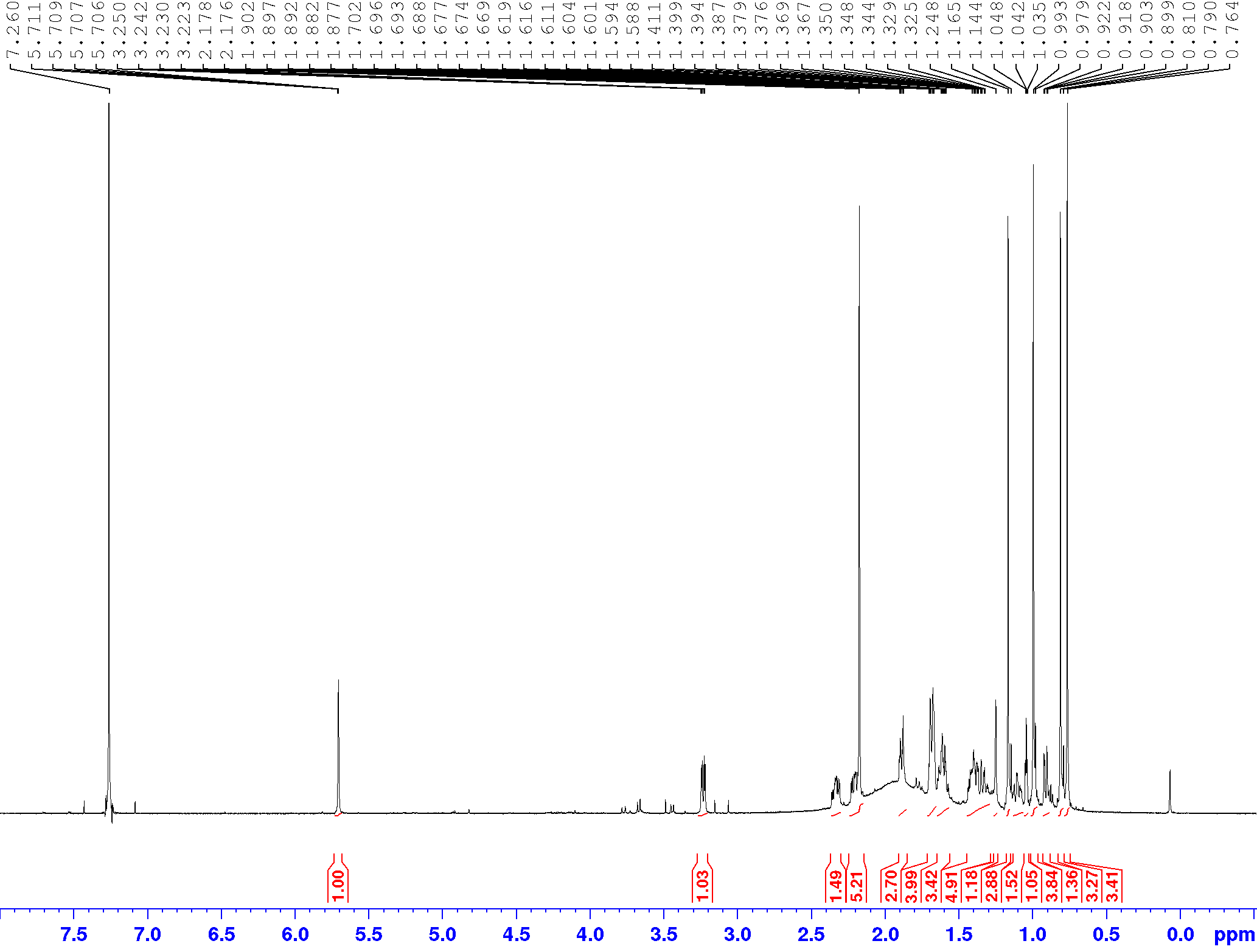


**S32.** ^13^C NMR (150.9 MHz, CDCl_3_) spectrum of Compound **8**.


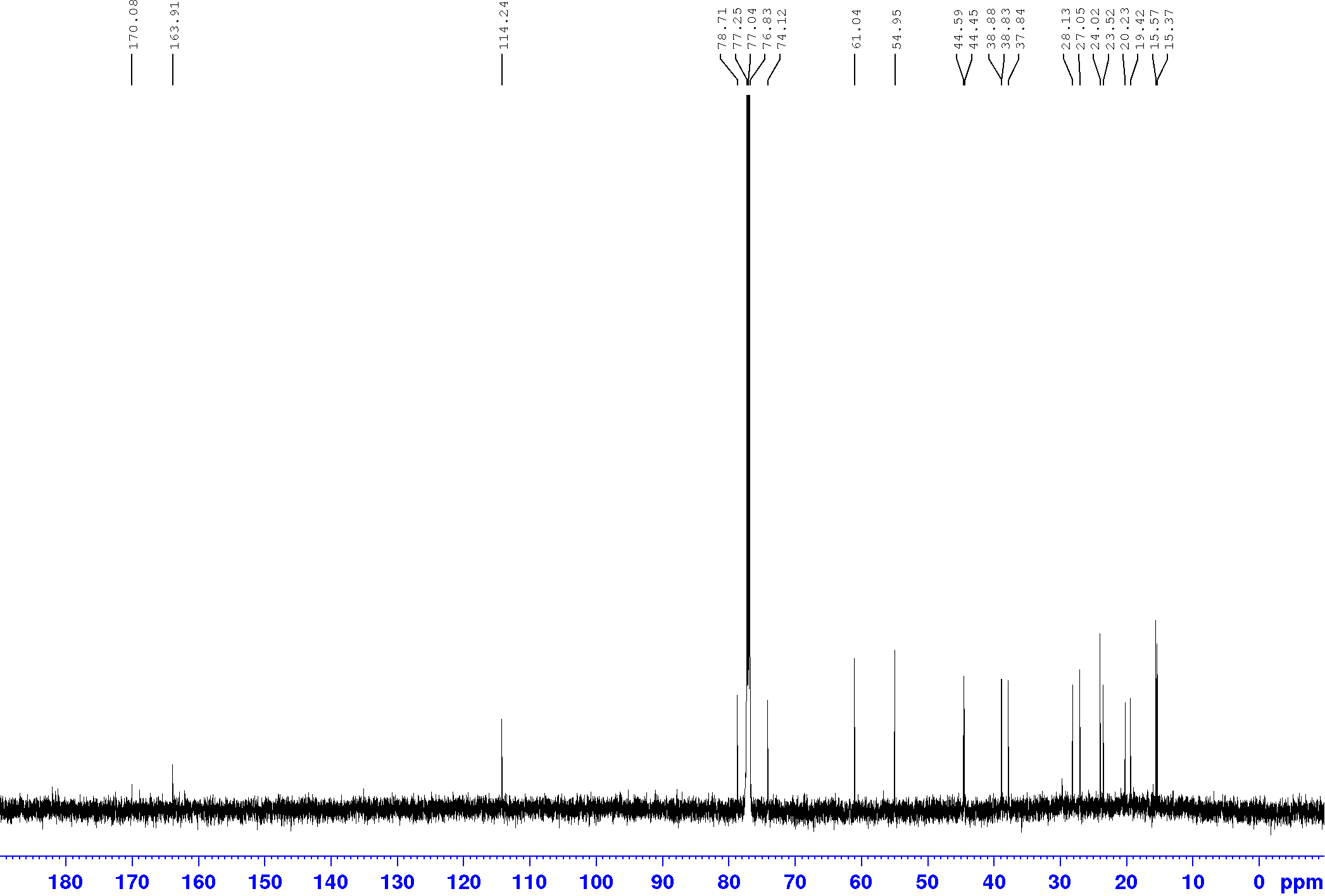


**S33.** HSQC (600.13 MHz, CDCl_3_) spectrum of Compound **8**.

**
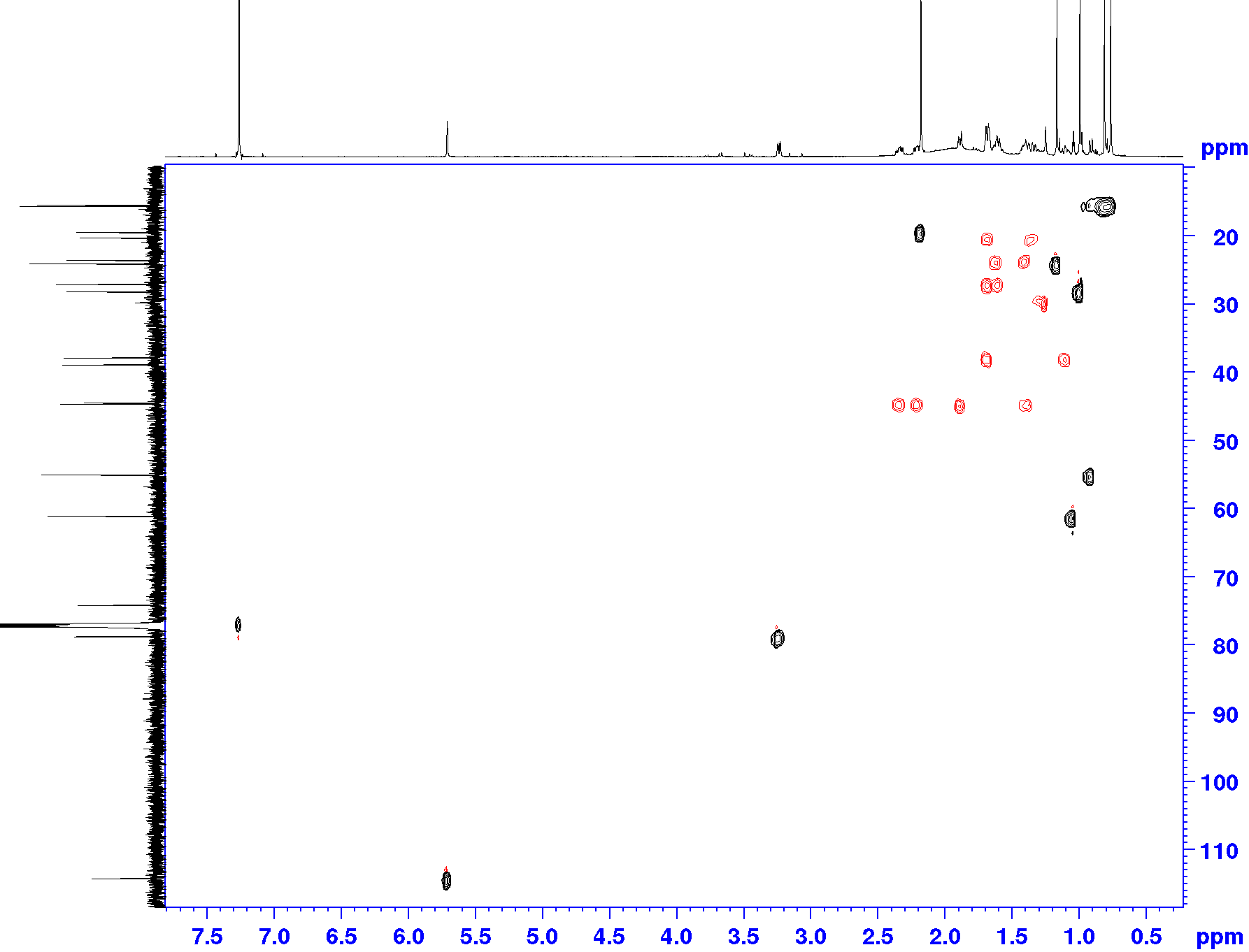
**

**S34.** HMBC (600.13 MHz, CDCl_3_) spectrum of Compound **8**.


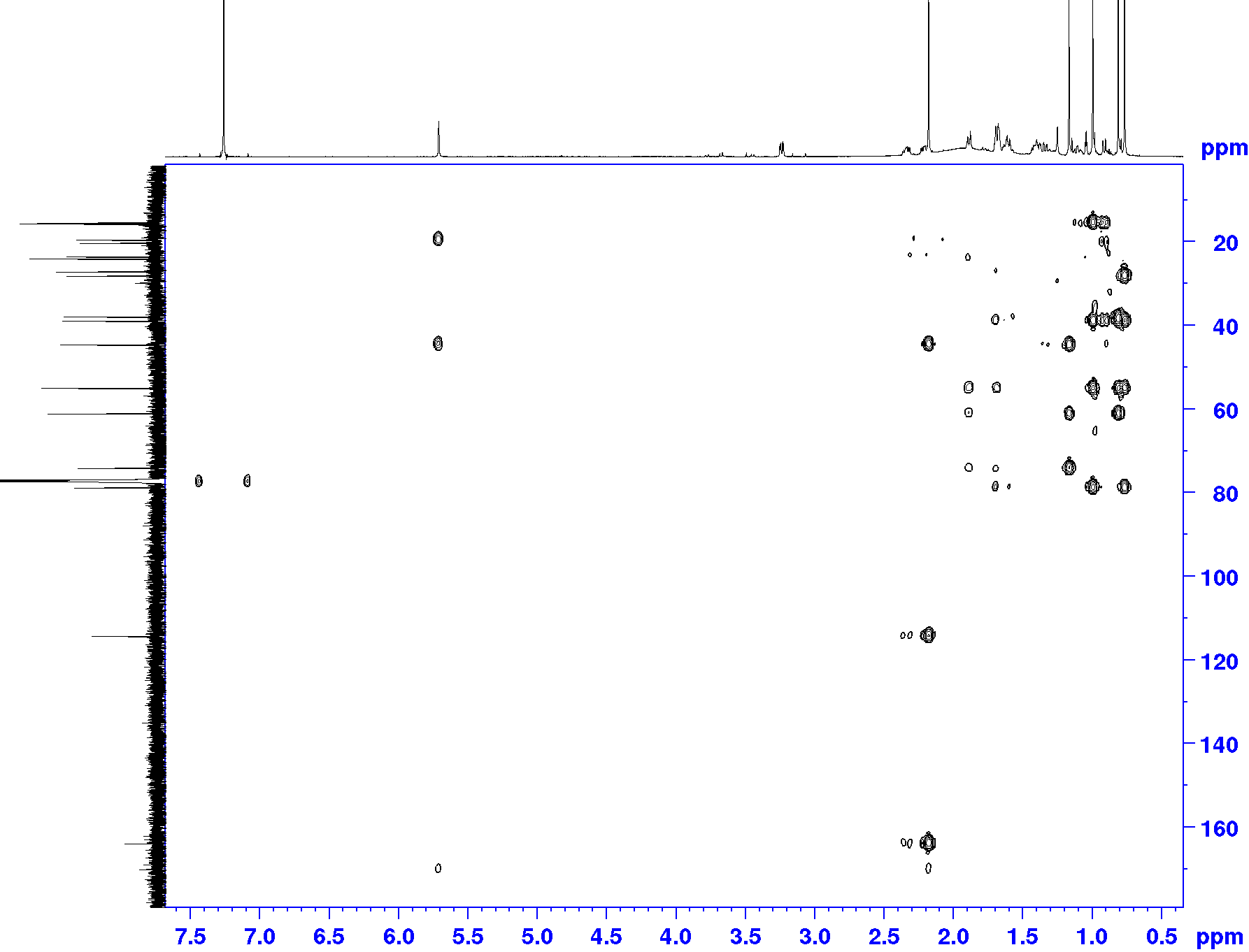

Supplement: NMR and MS data of biotransformation products. [file rsos170854supp1.docx]
